# *Cis* P-tau is a central circulating and placental etiologic driver and therapeutic target of preeclampsia

**DOI:** 10.1038/s41467-023-41144-6

**Published:** 2023-09-05

**Authors:** Sukanta Jash, Sayani Banerjee, Shibin Cheng, Bin Wang, Chenxi Qiu, Asami Kondo, Jan Ernerudh, Xiao Zhen Zhou, Kun Ping Lu, Surendra Sharma

**Affiliations:** 1 Departments of Pediatrics, Women and Infants Hospital, Warren Alpert Medical School, Brown University, Providence, RI 02905 USA; 2 Division of Translational Therapeutics, Department of Medicine, Beth Israel Deaconess Medical Center, Harvard Medical School, Boston, MA 02215 USA; 3 Department of Biomedical and Clinical Sciences, Linköping University, SE 58183 Linköping, Sweden; 4 Department of Clinical Immunology and Transfusion Medicine, Linköping University, SE 58183 Linköping, Sweden; 5 Departments of Biochemistry, Schulich School of Medicine & Dentistry, Western University, London, ON N6G 2V4 Canada; 6 Departments of Oncology, Schulich School of Medicine & Dentistry, Western University, London, ON N6G 2V4 Canada; 7 Departments of Pathology & Laboratory Medicine, Schulich School of Medicine & Dentistry, Western University, London, ON N6G 2V4 Canada; 8 Lawson Health Research Institute, Schulich School of Medicine & Dentistry, Western University, London, ON N6G 2V4 Canada; 9 Robarts Research Institute, Schulich School of Medicine & Dentistry Western University, London, ON N6G 2V4 Canada; 10 Departments of Pathology, Women and Infants Hospital, Warren Alpert Medical School, Brown University, Providence, RI 02905 USA

**Keywords:** Phosphorylation, Target identification, Protein aggregation, Pre-eclampsia

## Abstract

Preeclampsia (PE) is the leading cause of maternal and fetal mortality globally and may trigger dementia later in life in mothers and their offspring. However, the etiological drivers remain elusive. *Cis* P-tau is an early etiological driver and blood biomarker in pre-clinical Alzheimer’s and after vascular or traumatic brain injury, which can be targeted by stereo-specific antibody, with clinical trials ongoing. Here we find significant *cis* P-tau in the placenta and serum of PE patients, and in primary human trophoblasts exposed to hypoxia or sera from PE patients due to Pin1 inactivation. Depletion of *cis* P-tau from PE patient sera by the antibody prevents their ability to disrupt trophoblast invasion and endovascular activity and to cause the PE-like pathological and clinical features in pregnant humanized tau mice. Our studies uncover that *cis* P-tau is a central circulating etiological driver and its stereo-specific antibody is valuable for early PE diagnosis and treatment.

## Abstract (web summary)

Preeclampsia is the leading cause of maternal and fetal mortality worldwide. Here, the authors show that *cis* P-tau is a central circulating etiologic driver in preeclampsia and that the stereo-specific antibody targeting *cis* P-tau holds promise for early diagnosis and treatment of the disease.

## Introduction

As the leading cause of global maternal and perinatal morbidities and mortality, preeclampsia (PE) is a multi-system pregnancy-specific disorder diagnosed in 5–8% of all pregnant women^1–4^. This progressive disorder is characterized by de novo onset of hypertension and proteinuria or the new onset of hypertension plus significant end-organ dysfunction with or without proteinuria typically presenting after 20 weeks of gestation or postpartum^1–4^. It causes approximately 70,000 deaths annually^4^ and its prevalence continues to increase^5^. This disorder is believed to arise from multi-factorial etiology impacting the placental and systemic organ functions^1–4^, with blood RNAs as new biomarkers^6–8^. PE represents a unique example of proteinopathy in a younger population that can also lead to subsequent chronic conditions later in life, including mild cognitive impairment and dementia in mothers^9–11^ and their offspring^12,13^. However, the early etiological drivers underlying PE and its link to dementia remain unknown.

Tau protein stabilizes and coordinates microtubule assembly primarily in neurons and is regulated by phosphorylation^14–16^. Tau phosphorylation decreases with aging^17^, but greatly increases in Alzheimer’s disease (AD)^18–22^ and chronic traumatic encephalopathy (CTE) associated with traumatic brain injury (TBI)^23–26^. Tau hyperphosphorylation disrupts its normal function and gains toxic function, prone to tau oligomerization, aggregation, and tangle formation^18–22^. Notably, tauopathy can spread in the brain like a prion^27–30^, but can be attenuated by immunotherapy, with some antibodies being evaluated in clinical trials^31–33^.

Notably, tau conformation and function are further regulated after phosphorylation by a unique prolyl *cis-trans* isomerase, Pin1^31^. As a stress responsive enzyme, Pin1 protects against age-dependent neurodegeneration by catalyzing *cis* to *trans* isomerization of the pThr231-Pro motif in tau, restoring its microtubule function and promoting its degradation and dephosphorylation^34–37^. However, Pin1 is inhibited in AD or after TBI or stroke by mechanisms such as oxidation^38,39^, phosphorylation^40,41^, cytoplasmic sequestration^34^ or even mutations^42^. Pin1 inactivation leads to P-tau231 accumulation in the pathogenic *cis* conformation. The resulting *cis* P-tau231 causes and propagates the neuropathological process called cistauosis, leading to neurodegeneration like a prion a long before or ever tangle formation in AD, CTE after TBI, and vascular dementia (VaD) after stroke^39,40,43^. Importantly, this disease driver can be specifically recognized and neutralized by stereo-specific monoclonal antibody (mAb); whose treatment not only targets *cis* P-tau for TRIM21-mediated proteasomal degradation, but also restores brain pathology and dysfunction in animal models of AD, TBI, or stroke^39,40,43^. Moreover, in humans, *cis* P-tau231 or P-tau231 in the blood is an early biomarker for incipient AD pathology^44^ and pre-clinical AD^45^ as well as acute and chronic TBI^46^, respectively. Of note, the *cis* P-tau mAb is the only tau drug candidate in clinical trials that is efficacious not only in AD but also in TBI and stroke^31–33^, which are among the most well-established risk factors for dementia^23–25,47,48^. Thus, the Pin1-tau balance is critical for physiological health of cells to counter cistauosis in response to stress^31^.

The question arises whether the Pin1-*cis* P-tau dysregulation occurs in any younger age disorders since phosphorylation and its conformational regulation are believed to be central in diverse cellular processes in health and disease^49–51^. Notably, preeclampsia (PE), a severe pregnancy complication, appears to be a proteinopathy-associated syndrome^52–55^. We have recently developed a novel blood test for PE and AD using an autophagy-deficient human trophoblast cellular model coupled with a fluorescent dye with unique binding affinity for aggregated proteins^56^. Importantly, PE is associated with impaired autophagy and compromised lysosomal biogenesis machinery in the placenta^55,57^. This led us to hypothesize that impaired autophagy promotes accumulation of protein aggregates in the placenta and circulation in PE patients. This assay detects significantly higher levels of protein aggregates made of transthyretin and amyloid β42 in serum from PE patients^56^. However, despite elevated tau in blood of PE patients^56,58–60^, it is currently unknown whether pathogenic tau such as cis P-tau is present in the PE placenta and serum, and whether tauopathy plays any role in the pathophysiology of PE.

To demonstrate the significance of tau phosphorylation in early- and late-onset PE, we used multiple approaches to show significantly elevated expression of *cis* P-tau and other hyperphosphorylated tau isoforms in PE placental tissues and sera. Using in vitro and in vivo approaches, we show that *Cis* P-tau and anti-angiogenic factors, sFlt-1 and soluble endoglin (sEng)^60,61^ were induced in primary human trophoblasts in response to hypoxia and sera from PE patients, along with disrupted trophoblast invasion and endovascular activity. Moreover, depletion of *cis* P-tau from PE sera almost fully prevented the ability of the PE sera to induce all the PE-like pathological and clinical features in pregnant humanized tau mice. Thus, *cis* P-tau is a central circulating etiological factor for PE and its gestational age-dependent detection could be developed for early diagnosis and treatment by targeting *cis* P-tau.

## Results

### Robust *cis* P-tau in human placenta from early- and late-onset PE deliveries

**Fig. 1:**
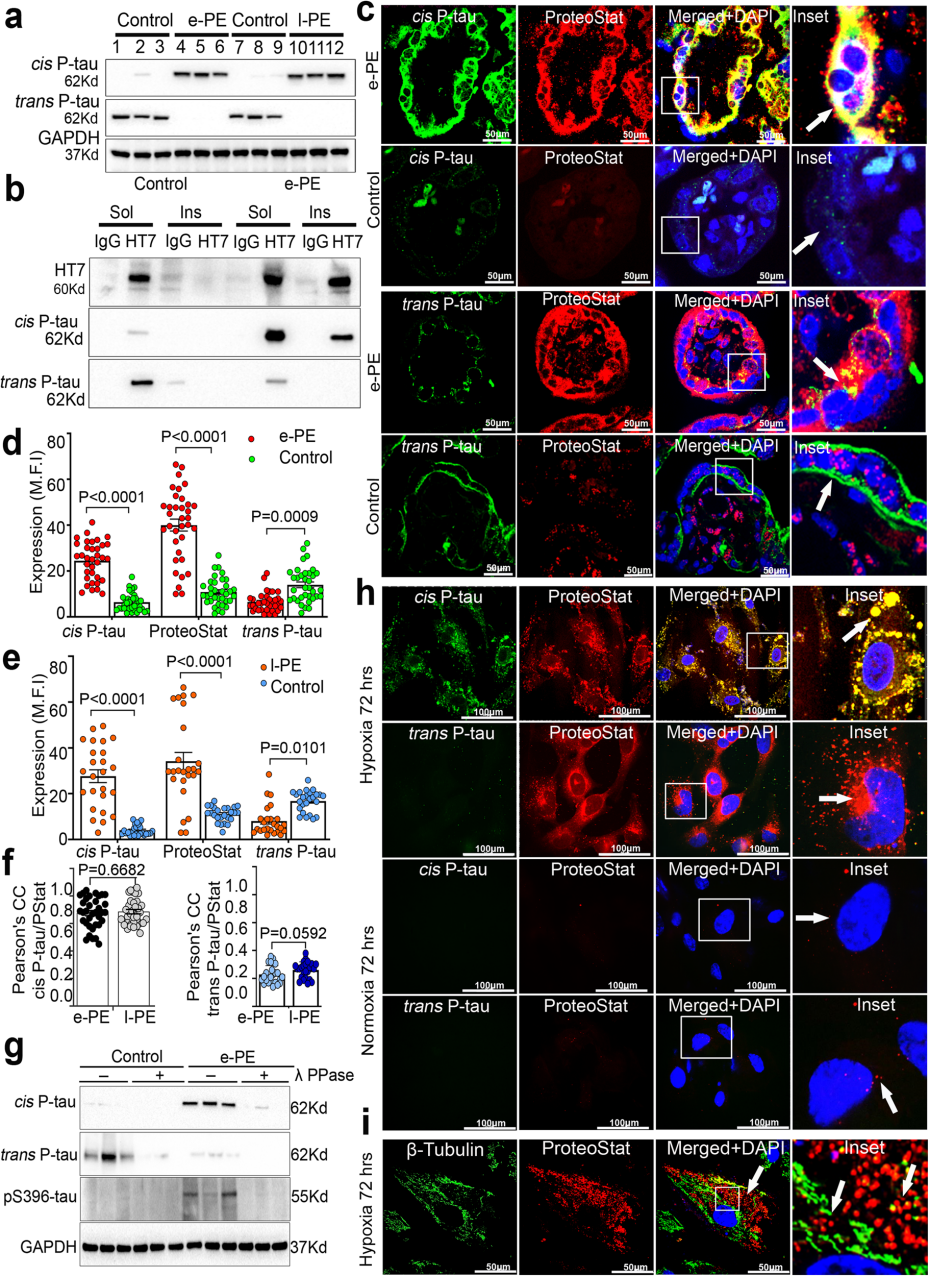
Robust *cis* P-tau expression in PE placenta and trophoblast cells exposed to hypoxia. **a** Immunoblots of placental protein extracts from gestational age-matched control (control; *n* = 3), early onset PE (e-PE; *n* = 3), and late onset PE (l-PE; *n* = 3) probed for *cis* and *trans* P-tau. **b** Sarkosyl soluble and insoluble fractions of placental extracts from age-matched controls (*n* = 7) and early-onset PE (e-PE; *n* = 7) were immunoprecipitated with HT7 (t-tau) and IgG antibodies before immunoblots against *cis* and *trans* P-tau. **c** Representative confocal images of human placental tissue sections from e-PE and age match control for double immunofluorescence with *cis* P-tau (green), *trans* P-tau (green), and ProteoStat (red). Inserts are magnified images of boxed areas. Cytotrophoblasts: complete arrow; syncytiotrophoblasts: incomplete arrows. Scale bar: 50 μm. Mean fluorescence intensity (MFI) quantification of *cis* P-tau, *trans* P-tau and ProteoStat positive protein aggregates in e-PE (**d**) and l-PE (**e**) placental sections (e-PE, *n* = 17; control, *n* = 16; l-PE, *n* = 19; control, *n* = 21, total 33–40 placental sections were analyzed per condition, two way ANOVA followed by Bonferroni’s post hoc test; mean±s.e.m. **f** Pearson’s colocalization coefficient (PCC) for *cis* P-tau and ProteoStat (left) is higher than for *trans* P-tau and ProteoStat (right); *n* = 35. Statistics are represented in the figure derived from two-tailed unpaired *t* test with Mann Whitney test.; mean±s.e.m. **g** Age-matched (control) and e-PE placental lysates were treated with (+λ) or without (-λ) lambda phosphatase (Ppase), followed by immunoblot with antibodies identifying early (*cis* P-tau and *trans* P-tau) or late (pS396) tau phosphorylation. Loading control was GAPDH. **h** Hypoxia-reoxygenation (H/R) stress induces robust *cis* P-tau aggregation. Human primary trophoblast cells were exposed to hypoxia (1% O_2_) for 72 h followed by 2-3 h incubation in reoxygenation (21% O_2_) conditions or normoxia environment (21% O_2_). The merged picture with nuclear DAPI demonstrated co-localization. Inserts were enlargements of boxed portions. Images are representatives of *n* = 7 independent experiments, scale bar: 50 μm. **i** Disruption of β-tubulin positive microtubule network due to hypoxia in human primary trophoblast cells. ProteoStat positive protein aggregate puncta accumulated near damaged microtubule structure. Images are representatives of *n* = 5 independent experiments. Bar: 100 μm.

Given no reference point for *cis* P-tau231 in the placenta, we examined its presence, along with that of physiological *trans* P-tau and total tau (t-tau) in placental tissues from normal pregnancy and PE deliveries using several complementary approaches (Fig. 1a–f). *Cis* P-tau was detected in early- (e-PE) and late (l-PE)-onset PE placental tissues (e-PE, *n* = 7; l-PE, *n* = 10), but not in an equal number of gestational age-matched controls, whereas *trans* P-tau was mainly present in control placenta (Fig. 1a, and Supplementary Figs. 1a–f, 2a, b). The demographic data and clinical characteristics of the PE patients used are presented in Supplementary Fig. 3a, b.

*Cis*, but not *trans*, P-tau231 is considered as the early driver of neurodegeneration^31,37,39,40,44,46^ and disease-associated tauopathy involves significant increase in insoluble hyperphosphorylated tau protein in the form of aggregates^62,63^. Using the Sarkosyl fractionation, followed by immunoprecipitation and immunoblotting^36^, we examined soluble and insoluble features and protein aggregation propensity of *cis* and *trans* P-tau in PE (e-PE; *n* = 8) and control (*n* = 12) placental tissues. *Trans* P-tau and weak *cis* P-tau were mainly present in the soluble form in control placenta (Fig. 1b). In contrast, *cis* P-tau, but not *trans* P-tau, was present in the insoluble form in e-PE placenta (Fig. 1b), suggesting the aggregated nature of *cis* P-tau.

We next examined the localization of *cis* P-tau in the e-PE and l-PE placental tissues by immunostaining. Since trophoblast localization of pathogenic *cis* P-tau is likely to be key to induce placental insufficiency, it is important to refer to the morphological details of the placental villous structure, depicting the respective localization of syncytiotrophoblasts (STB), cytotrophoblasts (CTB), extravillous trophoblasts (EVT), and fetal artery in the villous structure (Supplementary Fig. 2c). We have recently developed protein aggregate detection assay by employing a rotor dye, ProteoStat^TM^, which efficiently binds to the β-sheet structure of the aggregated proteins^56^. Figure 1c shows respective *cis* or *trans* P-tau immunofluorescence staining in e-PE placental tissues (*n* = 17) with different regions from each placenta (total *n* = 34–40). A strong *cis* P-tau signal mainly localized to the trophoblast layer in the villous structure and co-localized strongly with the ProteoStat signal (Fig. 1c), supporting the aggregated nature of *cis* P-tau. In contrast, *trans* P-tau was poorly present in the e-PE placenta but showed significant signal in the trophoblast layer of the control placenta (Fig. 1c). Specific ProteoStat staining was mainly detected in the e-PE placenta, but such ProteoStat staining was significantly reduced in control placenta, supporting the inability of physiological *trans* P-tau to aggregate^37,39^. These results are further confirmed by quantifying the mean fluorescence intensity (MFI) of immunostaining signals in all placental tissues (Fig. 1d, e). A question may arise whether significant *cis* P-tau signal compared to that for *trans* P-tau in the PE placenta is due to lack of co-localization with t-tau (HT7). We showed that t-tau equally co-localized with *cis* P-tau and *trans* P-tau, supported by Pearson correction coefficient (PCC) analysis (Fig. 1f and Supplementary Fig. 2d, e). Additional e-PE and l-PE placental tissues further confirmed robust *cis* P-tau staining in the trophoblast layer of the placental villi, whereas *trans* P-tau was poorly detected (Supplementary Fig. 4a, b).

To determine whether PE is also associated with tau hyperphosphorylation, placental extracts from e-PE and age-matched controls were incubated with lambda phosphatase (λ PPase) and then immunoblotted for early (*cis* P-tau) and late (pS396) tau phosphorylation markers. Both *cis* P-tau and pS396-tau were detected in PE and their signals were greatly reduced by λ PPase (Fig. 1g), confirming their phosphorylated status. In contrast, physiological *trans* P-tau was significantly expressed only in control tissues but was equally dephosphorylated by λ PPase. Thus, placental tau in PE is highly prone to *cis* P-tau-like hyperphosphorylation and aggregation.

### Hypoxia induces *cis* P-tau in primary human trophoblasts

The endoplasmic reticulum (ER) stress induced by hypoxia or other danger signals is thought to be a key contributor to placental insufficiency^64–66^. We next investigated whether hypoxia (1%) coupled with transient reoxygenation (H/R) induced protein aggregation, including that of *cis* P-tau, in primary human trophoblasts (PHTs). PHTs were exposed to H/R or normoxia for 72 h, followed by staining with antibodies for *cis* P-tau or *trans* P-tau and ProteoStat. H/R potently induced *cis* P-tau, but not *trans* P-tau, aggregation (Fig. 1h). Their kinetic appearance is shown in Supplementary Fig. 5. Normoxia failed to induce either *cis* P-tau or protein aggregation even after 72 h of incubation (Fig. 1h, and Supplementary Fig. 5a, b). However, investigation of spatiotemporal dynamics of *cis* P-tau induction during H/R and its correlation with protein aggregate accumulation reveals that *cis* P-tau could be induced as early as 6 h after hypoxia exposure, but surprisingly, no ProteoStat signal was observed at this time point (Fig. 1h, and Supplementary Fig. 5a, c). *Cis* P-tau induction further intensified at later time points peaking at 48–72 h interval. Interestingly, *cis* P-tau induction was accompanied by protein aggregation starting at 24 h and becoming intense by 48 and 72 h time points. Co-localization of *cis* P-tau and ProteoStat was amply evident at these times points as shown in inset panels (Fig. 1h and Supplementary Fig. 5a). Since *cis* P-tau disrupts microtubule assembly in neurons^31,37,39,40^, to better understand the impact of *cis* P-tau on the microtubule organization in human trophoblasts, we performed a time-course analysis of β-tubulin and *cis* P-tau co-localization in hypoxia-exposed PHTs. 72 h Hypoxia resulted in the disassembly of microtubules and the formation of patch-like protein aggregates (Fig. 1i, white solid arrow). Moreover, β-tubulin was disorganized and co-localized with *cis* P-tau at even 24 h of incubation (Supplementary Fig. 6a), suggesting its compromised role in microtubule assembly in hypoxia-exposed PHTs. In contrast, even after 72 h exposure to normoxia, tubulin in PHTs remained properly organized and no ProteoStat signal was induced (Supplementary Fig. 6b). To assess the endurance of hypoxia-induced *cis* P-tau in PHTs, we performed a chase experiment where 72 h hypoxia-exposed PHTs were incubated under normoxia conditions. *Cis* P-tau abundance declined after 48 h chase incubation (Supplementary Fig. 6c). We hypothesize that hypoxia-induced ER stress causes significant induction of *cis* P-tau prior to protein aggregation in PHTs, as shown in stressed neurons^39^. We have previously published that the PE placenta and hypoxia (ER inducer)-exposed PHTs show the features of ER stress, sterile inflammation/pyroptosis and impaired autophagy^65,66^. To investigate the role of ER stress for *cis* P-tau accumulation, we incubated PHTs with ER stress inducer tunicamycin at 2.5 µg/ml for 6 h. Tunicamycin, even at this comparatively low concentration, caused significant *cis* P-tau accumulation when compared to the vehicle control (Supplementary Fig. 6d). To investigate whether ER stress acts downstream of hypoxia in the *cis* P-tau accumulation, we pretreated the cells with the inhibitor (4μ8c) of the inositol-requiring enzyme type1 (IRE1), a key regulator of the unfolded protein response (UPR), for 3 h prior to hypoxic exposure for 16 h. Preincubation with 4μ8c significantly reduced hypoxia-induced *cis* P-tau accumulation (Supplementary Fig. 6e). These results demonstrate that hypoxia induces *cis* P-tau in primary human trophoblasts, as shown in the neurons^39^.

### Colocalization of *cis* P-tau with other early hyperphosphorylated tau isoforms

Since *cis* P-tau is a precursor of tauopathy^31,37,39,40,44,46^, we next analyzed its relationships with other tau isoforms in the hierarchical pattern of tau phosphorylation^67^ in placental tissues from e-PE and l-PE deliveries using serine/threonine-proline motifs-directed mAbs. *Cis* P-tau immunostaining signal was prominent in the trophoblast layer, particularly syncytiotrophoblasts in PE, but not in controls (Supplementary Figs. 7 and 8, e-PE, *n* = 7; l-PE, *n* = 8). All tau proteins with early or late appearance, pT231-tau (early), AT8-tau (intermediate), pS396 (late), and T22 oligomeric-tau, co-localized with *cis* P-tau, albeit in the trophoblast layer of the e-PE placenta (Supplementary Fig. 7). AT100-tau (associated with mature tangles) was the only tau isoform that was moderately expressed in the e-PE placenta and did not co-localize with *cis* P-tau. Similar data were also obtained with placental tissue from l-PE deliveries (Supplementary Fig. 8). To consider the colocalization across tau isoforms and their expression levels, we calculated the PCC between *cis* P-tau and other tau proteins at each data point to determine their colocalization patterns. The PCC was significantly higher (*r* ≥ 0.5) across the PE samples (both e-PE and l-PE) compared to age-matched controls (*r* = 0.05 or *r* = 0.2). A direct linear correlation (R^2^) between *cis* p-tau and pT231, AT8, pS396, and T22 in multiple PE placental tissue sections (*n* = 30) demonstrates that high *cis* P-tau levels were consistently associated with an increase in hyperphosphorylated tau species in the PE placenta, but not in age-matched controls (Supplementary Figs. 7 and 8). Notably, tau AT100 was only detected as small clusters in the e-PE, but not in l-PE, placenta and no linear correlation was detected between AT100 and *cis* P-tau expression in the e-PE placenta (Supplementary Figs. 7 and 8). Thus, *cis* P-tau is colocalized with other early hyperphosphorylated tau isoforms in the PE placenta.

### Pin1 inactivation induces *cis* P-tau in human PE placenta

**Fig. 2:**
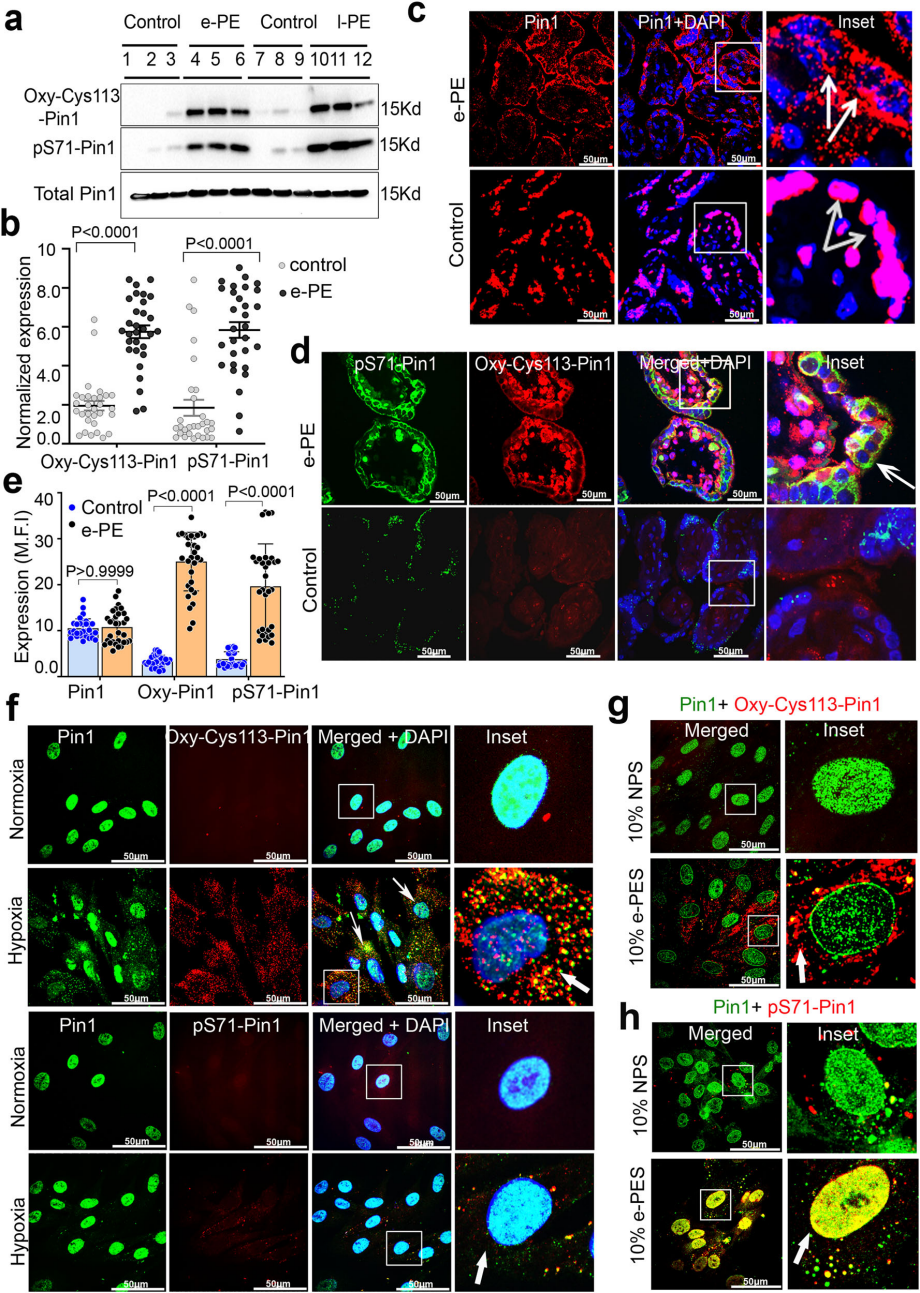
Robust Pin1 inhibitory post-translational modifications in PE placenta and primary trophoblast cells exposed to hypoxia or PES. **a** Placental protein extracts from age matched control, e-PE and l-PE (*n* = 3) were subjected to immunoblot to detect total Pin1 and inactive forms of Pin1 (Oxy-Cys113 Pin1 and pS71-Pin1). **b** Mean normalized expression of Oxy-Cys113 Pin1 and pS71-Pin1. Three separate placental segments were selected for analysis from each person (e-PE, *n* = 17; control, *n* = 16, two-way ANOVA followed by Sidak’s post hoc test; mean ± s.e.m). **c** Representative confocal images of human placental tissue sections from e-PE and age match control to probe total nuclear Pin1. The merged pictures with nuclear DAPI (and enlarged inset) demonstrated nuclear localization of total Pin1. Scale bar: 50 μm. **d** Human placental tissue sections from e-PE and gestational age match control were probed for double immunofluorescence with oxidized Pin1 (Oxy-Cys113 Pin1, red) and phospho-Pin1 (pS71-Pin1, green) and then subjected to confocal imaging. pS71-Pin1 and Oxy-Cys113Pin1 significantly concentrated at the Cyto and syncytotrophoblast layers of the human placenta. Pin1 inactivation by oxidation (Oxy-Cys113 Pin1) is more prominent than phosphorylation (pS71-Pin1). Inactive Pin1 forms are localized outside DAPI positive nucleus. Scale bar: 50 μm. **e** Mean fluorescence intensity (M.F.I) quantification of Pin1, Oxy-Cys113 Pin1 and pS71-Pin1 in placental sections from e-PE and age matched control (e-PE, *n* = 17; control, *n* = 16, total 27–35 placental sections were analyzed per condition, two way ANOVA followed by Bonferroni’s post hoc test; mean ± s.e.m. **f**, **g** Hypoxia induced Pin1 Cys113 oxidation inhibits its catalytic activity and impairs Pin1 subcellular localization. Human primary trophoblast cells were exposed to normoxia (21% O_2_) or hypoxic stress (1% O_2_) for 24 h followed by double immunofluorescence with (**f**) anti-Pin1 (green) and anti-Oxy-Cys113-Pin1 (red) or (**g**) anti-Pin1 (green) and anti-pS71-Pin1 antibodies. The merged picture with nuclear DAPI (and enlarged inset) showing colocalization. Hypoxia reduces Pin1 nuclear localization and increases cytoplasmic localization. Images are representatives of *n* = 7 independent experiments. Scale bar: 50 μm. **h** Robust induction of Pin1 inactive forms (Oxy-Cys113 Pin1 and pS71-Pin1) at 24 h following incubation of primary trophoblast cells with PES (*n* = 5), but not NPS (*n* = 5). Confocal Images are representatives of *n* = 5 independent experiments. Scale bar: 50 μm.

The above results indicate that tauopathy-promoting tau isoforms are robustly induced in the PE placenta. The unique isomerase Pin1 isomerizes the pThr231-Pro motif in tau to restore tau microtubule function and to prevent the accumulation of pathogenic *cis* tau isoforms^31,37,39^. Notably, Pin1 is known to be catalytically inactivated by multiple mechanisms, including Cys113 oxidation^38,39^ and Ser71 phosphorylation^40,41,68^, resulting in the cytoplasmic sequestration^34,38,68^. We determined if Pin1 was inactivated by these mechanisms by evaluating PE placental tissues, hypoxia-exposed PHTs, and PE sera-exposed PHTs. Indeed, oxidized Oxy-Cys113 Pin1 and phosphorylated pS71-Pin1 were readily detected in placental tissues from e-PE (*n* = 7) and l-PE (*n* = 10), as compared to controls (Fig. 2a, b, and Supplementary Fig. 9a, b), consistent with *cis* P-tau induction (Fig. 1a, and Supplementary Figs. 1–6). Interestingly, Pin1 was mainly nuclear in control tissues, as shown^34^, but was diffusely cytoplasmic in PE placenta, despite no significant change in total Pin1 (Fig. 2a, b, and Supplementary Fig. 9a, b). These data support Pin1 post-translational modifications, not downregulation, as the key mechanisms for its inactivation^34,38,40,41^. Increased Pin1 oxidation and phosphorylation were further confirmed by immunohistochemical staining of PE placental tissue (*n* = 5) (Fig. 2c, d, e, and Supplementary Fig. 9c). The z-stack reconstruction of the confocal images showed robust pS71-Pin1 and oxy-Cys113-Pin1 immunoreactivity throughout STB and CTB in all the e-PE placental sections, whereas control placental tissue contained weak and very diffuse signals (Fig. 2d, Supplementary Fig. 9c insets, arrow).

Since hypoxia induces *cis* P-tau in PHTs, we examined whether hypoxia also influenced post-translation modifications of Pin1, as seen in PE placental tissues. Under normoxic conditions, Pin1 was mainly localized to the nucleus and there was no evidence of Oxy-Cys113-Pin1 and pS71-Pin1 (Fig. 2f, and Supplementary Fig. 9d). However, hypoxia treatment of PHTs significantly induced non-nuclear form of Oxy-Cys113-Pin1, with moderate induction of pS71-Pin1 (Fig. 2f). The latter observation is different from those in the PE placental tissues, suggesting that the placental tissues most probably experience additional pathological events in addition to hypoxic microenvironment. To confirm this possibility, we used serum samples from PE patients (PES) and normal pregnancy (NPS) to assess their ability to induce post-translational changes in Pin1 in PHTs. After incubating PHTs with 10% (v/v) NPS or PES for 24 h, PES, but not NPS, significantly induced both Oxy-Cys113-Pin1 and pS71-Pin1 isoforms (Fig. 2g, h and Supplementary Fig. 9e, f), supporting that PES contains PE-inducing factors. Since DAPK1 phosphorylates Pin1 on S71 and is elevated in AD and after TBI or stroke^40,41,68,69^, we examined the changes of DAPK1 protein levels and the effects of DAPK1 inhibition in PHTs exposed to hypoxia or PE sera. When PHTs were treated for 24 h with hypoxia or PES, we found that DAPK1 was significantly elevated, while pS289-DAPK1 was reduced (Supplementary Fig. 10a, b), which has been shown to inhibit DAPK1 cellular function^70^. Moreover, inhibition of DAPK1 by an ATP-competitive inhibitor TC-DAPK6^71^ in PHTs significantly reduced *cis* P-tau accumulation in response to hypoxic stress (Supplementary Fig. 10c). Finally, Pin1 inactivation in PE was further supported by the demonstrations that HP1α was significantly reduced in hypoxia-exposed PHTs (Supplementary Fig. 10d) and in e-PE placenta as compared with age-matched controls (Supplementary Fig. 10e, f), as shown previously in AD, where reducing Pin1 activity leads to lower HP1α protein levels^72^. Moreover, our immunoprecipitation followed by immunoblotting analyses showed that Pin1 was associated with *cis* P-tau in e-PE placenta, but not in control placenta (Supplementary Fig. 10g), as report previously in AD neurons, where Pin1 is associated with pT231-tau^34^. These results together demonstrate that the induction of *cis* P-tau in human PE placenta is likely due to Pin1 inactivation.

### Inhibition of Pin1 not only induces *cis* P-tau, but also causes the onset of the PE-like features in pregnant humanized tau mice

We next examine whether Pin1 inhibition is sufficient to induce *cis* P-tau and PE-like characteristics in vitro and in vivo. To this end, we used Sulfopin, a recently identified covalent Pin1-specific inhibitor that targets the functionally critically Cys113 and the ATO-binding pocket in the Pin1 catalytic active site and effectively inhibits Pin1 function in cancer in vitro and in vivo^73,74^. Indeed, the inhibition of Pin1 PHTs by Sulfopin led to accumulation of *cis* P-tau and protein aggregates. At a dose of 1 µM, Sulfopin treatment for 24 or 48 h resulted in significant *cis* P-tau- and ProteoStat-specific protein aggregates (Supplementary Fig. 10h).

To examine whether Pin1 inhibition can induce the PE-related characteristics, we performed pilot experiments to evaluate the effects of Sulfopin on pregnant humanized tau (hTau) mice, in which mice received daily doses of 20, 10, or 5 mg/kg injection from gestational day 9 (gd 9) through gd 15. In hTau mice, administration of Sulfopin at 20 or 10 mg/kg, respectively, induced significant fetal resorption and increased fetal mortality (Supplementary Fig. 11a). Accordingly, we used Sulfopin at a dose of 5 mg/kg and this dose did induce PE-like features and accumulation of *cis* P-tau protein aggregates in the junctional zone of the placenta without affecting fetal and maternal health. Based on our preliminary results, we also increased the interval between subsequent doses (Supplementary Fig. 11b). Mice were injected with either Sulfopin (5 mg/kg) or vehicle on gd 9, 12 and 15 (*n* = 4). Doppler ultrasound was used on gd 17 to track umbilical artery velocity, and on gd 19, animals were housed for 24 hrs in metabolic cages for urine collection for proteinuria analysis. Animals were euthanized on gd 19 to collect serum and tissues for further analysis. Confocal imaging of entire placental sections showed significant accumulation of *cis* P-tau at the junctional zone (Jz) and labyrinth (Lb) and the formation of protein aggregates (ProteoStat^+^ signal) in Sulfopin-treated mice but not in vehicle-treated mice (Supplementary Fig. 11c). Interestingly, there was a significant colocalization of *cis* P-tau and ProteoStat signals in the junctional zone of the placenta, suggesting induction and aggregation of *cis* P-tau. Importantly, *cis*-P-tau accumulation and protein aggregates in the placenta due to Pin1 inhibition were also accompanied with the PE-like pathological and clinical features, including fetal growth restriction (Supplementary Fig. 11d), proteinuria (Supplementary Fig. 11e), increased sFlt-1 and sEng production (Supplementary Fig. 11f, g). elevated blood pressure and reduced umbilical and uterine arterial systolic velocities as measured by Pulse-wave Doppler Ultrasound imaging recorded on gd 16.5 (Supplementary Fig. 11h, i). Collectively, these results show that inhibition of Pin1 by Sulfopin not only induces *cis* P-tau in primary human trophoblasts, but also causes the onset of the PE-like features in pregnant humanized tau mice.

### Hypoxia- and PES-induced *cis* P-tau promotes tau aggregation in trophoblasts and disrupts the crosstalk between extravillous trophoblasts and endothelial cells

**Fig. 3:**
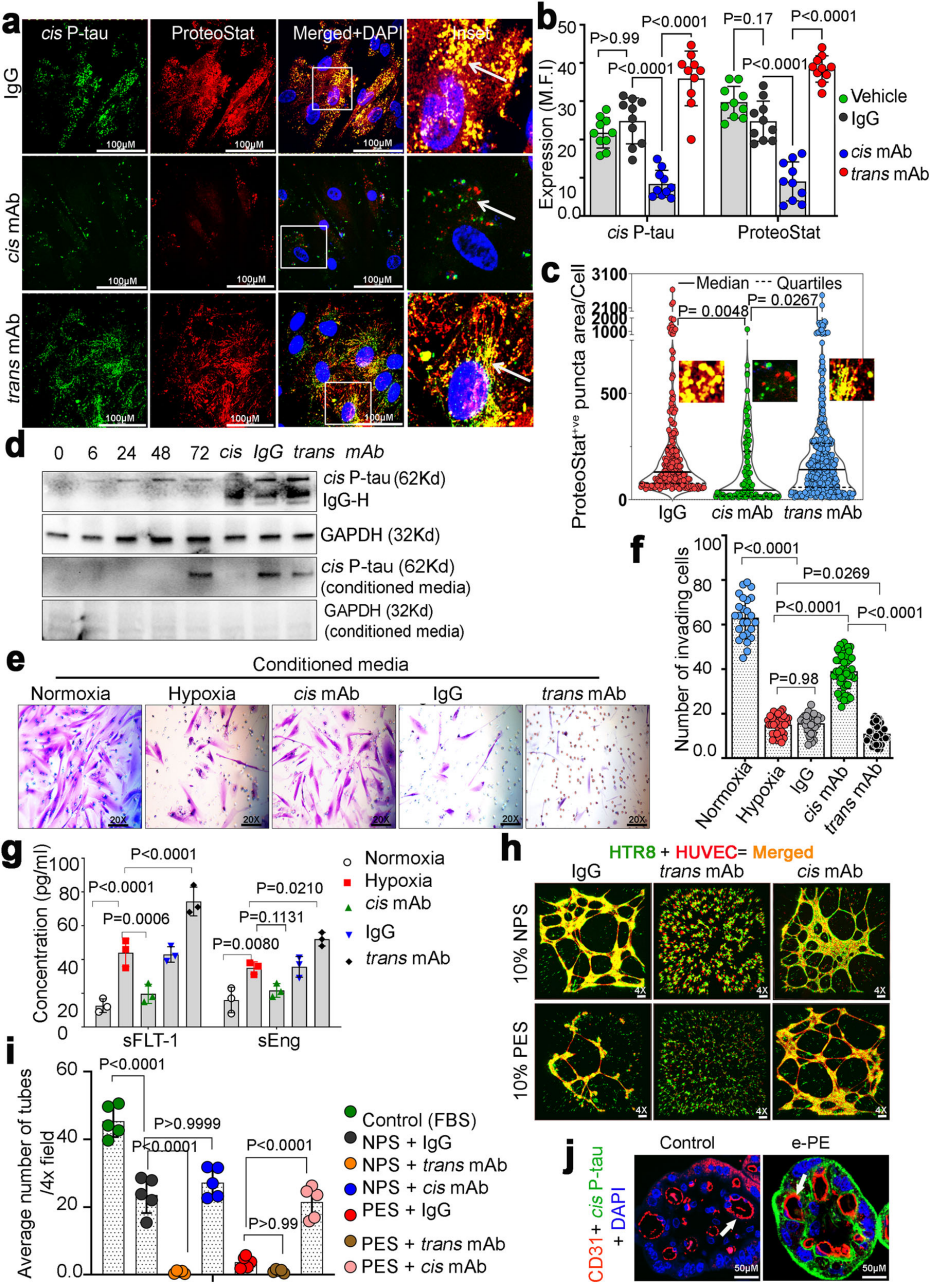
Hypoxia- and PES-induced *cis* P-tau promotes tau aggregation in trophoblasts and disrupts the crosstalk between extravillous trophoblasts and endothelial cells. **a**–**c** Human primary trophoblast cells were subjected to acute hypoxia for 72 h before being incubated for 24 h with *cis, trans*, or IgG isotype, followed by immunostaining with *cis* P-tau and ProteoStat (**a**), quantification of mean fluorescence intensity (**b**) and a violin plot to depict the abundance, distribution and area of ProteoStat positive protein aggregate puncta (**c**). Arrowhead points to either aggregation or disaggregation between *cis* P-tau and ProteoStat. *n* = 5, scale bar, 100 μm; two way ANOVA followed by Bonferroni’s post hoc test; mean±s.e.m. **b** 120 individual cells from each group were analyzed from *n* = 5 and statistics are represented derived from one way ANOVA followed by Tukey’s post hoc test; mean ± s.e.m. **c**, **d** Dynamic *cis* P-tau induction in trophoblast cells after acute hypoxia exposure. After 72 h of H/R exposure, cells were treated for another 24 h to the corresponding neutralizing antibody, followed by immunoblotting for *cis* P-tau (bottom panel). Condition media (CM) from the indicated time points as well as after indicated treatment with neutralizing antibodies were collected and concentrated, followed by immunoblot for *cis* P-tau and GAPDH. *n* = 5. **e** The effect of intracellular *cis*-P-tau accumulation and extracellular spread. Human trophoblast cells were seeded on the upper layer of the Matrigel coated membrane in the 3D Matrigel chamber and incubated with concentrated CM from experiments **a** and **d** for 24 and 48 h. The representative images were obtained after 24 h (*n* = 5) and show 20 x fields of Matrigel-coated 8 mm pore PET membranes after eliminating all noninvasive cells and staining the membrane with Crystal violet. **f** The percentage of cells migrating/invading through the Matrigel matrix to reach the underlying membrane is shown. Total 25–49 separate fields were analyzed from *n* = 5, one way ANOVA followed by Tukey’s post hoc test; mean±s.e.m. **g** CM from experiment **a** and **d** were analyzed by ELISA for the presence of soluble fms-like tyrosine kinase-1 (sFlt-1) and soluble endoglin (sEng). *n* = 3, two way ANOVA followed by Bonferroni’s post hoc test; mean±s.e.m. **h**, **i** CellTrackerTM red tagged HUVEC and CellTracker^TM^ green tagged HTR-8/SVneo were co-cultured overnight on Matrigel for in vitro 3D vascular tube development in the presence of NPS (10%) or e-PES (10% e-PES) supplemented with *cis*, *trans* or IgG isotype (10 μg/ml) (**h**). Tube-like structures created by coupled capillary bridges were counted in four fields to determine the average number of tubes/vacuoles formed (**i**). *n* = 7 different PES and NPS patients, two-way ANOVA followed by Bonferroni’s post hoc test; mean ± s.e.m. **j** Confocal images of single placental villi exhibiting juxtaposition of *cis* P-tau positive trophoblast layer and CD31 positive endothelial cells in human PE placenta (e-PE, *n* = 11; control, *n* = 8).

Given robust *cis* P-tau induction in the PE placenta and stressed PHTs, we asked whether such *cis* P-tau would affect tau deposition and aggregation in PHTs and disrupt endovascular cross talk between extravillous trophoblasts and endothelial cells. To this end, we first subjected PHTs to hypoxia for 72 h and then incubated for 24 h in the presence of control IgG or mAbs for *cis* or *trans* tau. *Cis*, but not *trans*, P-tau mAb effectively disrupted *cis* P-tau induction and aggregation (Fig. 3a). Rather, *trans* mAb appeared to exacerbate *cis* P-tau induction and protein aggregation, suggesting that residual *trans* P-tau did provide partial protection against *cis* P-tau (Fig. 3a, b). Analysis of spatio-temporal distribution of protein aggregates (size and number) by violin plot analysis also revealed that *cis* mAb-treated cells had significantly fewer or no large aggregates, whereas elimination of remaining *trans* P-tau increases the numbers and the median size of the aggregates (Fig. 3c). We also identified the functional significance of intracellular *cis* P-tau neutralization by performing immunoblot analysis of cell extracts and supernatant from time-dependent hypoxia-exposed PHTs (Fig. 3d). Although intracellular *cis* P-tau appeared early, a significant amount of *cis* P-tau was only detected in conditioned media after 72 h of stress, suggesting that extracellular *cis* P-tau spreading might occur after it reached a critical threshold intracellularly. Extracellular *cis* P-tau release was significantly blocked after intracellular *cis* P-tau elimination by *cis* mAb, but not by IgG isotype. By contrast, *trans* mAb further potentiated intracellular *cis* P-tau induction and spreading (Fig. 3b), which was further supported by the PCC calculations (Supplementary Fig. 12a). *Cis* P-tau mAb treatment did not induce any detectable cell death in PHTs measured by the lactate dehydrogenase release assay (Supplementary Fig. 12b), which monitors cell membrane leakage in dying cells^75^. Notably, elimination of *trans* P-tau using the *trans* mAb does not protect against, but rather enhances *cis* P-tau toxicity in stressed neurons^39^.

The above results have shown that *cis* P-tau is induced and spreads in a time-dependent manner in PHTs in response to stress. To examine how *cis* P-tau would propagate as proteopathic seed^39,40^ and how toxic *cis* P-tau seeding into unaffected trophoblast cells might be detrimental for its invasion, we assayed production of anti-angiogenic factors such as sFlt-1 and sEng and endovascular activity. PHTs were exposed to normoxia or hypoxia for 72 h, followed by incubation of control IgG, *cis* mAb or *trans* mAb for 24 h. Conditioned media were collected and added to fresh primary trophoblasts seeded on the upper layer of the Matrigel-coated membrane in a 3D Matrigel chamber for 24 and 48 h (Fig. 3e, and Supplementary Fig. 12c). Cells exposed to the conditioned media from normoxia showed significant invasion (24 h, *p* < 0.001 and 48 h, *p* < 0.001) towards the chemoattractant in the bottom chamber (Fig. 3e, f). In sharp contrast, cells exposed to the hypoxic conditioned media failed to invade/migrate towards the chemoattractant, which was restored by the conditioned media from *cis* mAb-treated cells, but not from IgG or *trans* mAb treated cells (Fig. 3e, f, and Supplementary Fig. 12c, d). Instead, conditioned medium from *trans* mAb-treated cells abrogated the invasion/migration. Thus, *cis* P-tau might propagate as toxic tau seed from one trophoblast to another and abrogate their migration and invasion capability, as shown in neurons^39,40^.

Production of antiangiogenic factors such as sFlt-1 and sEng has been associated with the PE pathophysiology^61,64,76^ and can be induced in human trophoblasts in vitro by hypoxia^77^. Indeed, hypoxia did induce sFlt-1 and sEng in PHTs, and importantly, their production was effectively blocked by *cis* Ab, not IgG or *trans* Ab (Fig. 3g). Rather, *trans* Ab had the opposite effects, inducing higher production of these factors compared to hypoxia alone (Fig. 3g). Additionally, our findings show that hypoxia causes a significant increase in transcription of alternatively spliced sFlt-1 variants in PHTs, which is significantly reversed by *cis* mAb treatment (Supplementary Fig. 12e). In contrast, *cis* mAb treatment of hypoxia-exposed PHTs had no impact on endoglin (Eng) mRNA expression (Supplementary Fig. 12e).

As discussed earlier, Pin1 inactivation by Sulfopin induces *cis* P-tau and its aggregation in pregnant hTau mice and in PHTs. A question arises whether Pin1 deficiency will lead to upregulated transcription of sFlt1 and endoglin, the anti-angiogenic factors associated with PE^61,64,76^. To assess the effects of Pin1 on FLT-1 alternative splicing, we exposed PHTs to Sulfopin in a time- and dose- dependent manner, followed by assaying FLT-1 alternative splicing using splicing variant-specific primers. Sulfopin appeared to induce FLT-1 alternative splicing in a time-dependent manner (Supplementary Fig. 12f), and less so in a dose-dependent manner (Supplementary Fig. 12g). These findings imply that protein aggregate formation involving *cis* P-tau may be partially necessary for significant alternative sFlt-1splicing.

We have previously demonstrated that PES disrupts endovascular cross talk between first trimester extravillous trophoblast cells (HTR8) and primary human umbilical vein endothelial cells (HUVECs)^52,78^. Notably, HTR8 cells alone do not form three-dimensional capillary-like tubes on Matrigel, but can interact with HUVEC to form the three-dimensional structures via their endovascular potential. To evaluate whether *cis* mAb could inhibit PES-induced disruption of three-dimensional capillary-like tube formation, we repeated the tube formation between HTR8 cells and HUVECs on Matrigel and confirmed our previous results (Supplementary Fig. 13a)^78^. Importantly, PES-induced disruption of three-dimensional capillary-like tube formation was restored by *cis* mAb, not IgG or *trans* mAb (Fig. 3h, i). To further examine the relationship between *cis* P-tau-positive trophoblasts and CD31-positive endothelial cells, we determined the localizations of *cis* P-tau and CD31, a marker for endothelial cells, in the blood vessels and the trophoblast layer in PE placental tissues. CD31 was prominently present on the surface of blood vessels, whereas *cis* P-tau was uniquely localized to the trophoblast layer (Fig. 3j, and Supplementary Fig. 13b), suggesting that *cis* P-tau may mainly disrupt trophoblast functions and their interaction with endothelial cells. These results together support our premise that *cis* P-tau in PES is detrimental to endovascular crosstalk between extravillous trophoblasts and endothelial cells, which is required for spiral artery remodeling^79,80^. It is not only the intrinsic expression of *cis* P-tau in the placenta but also its presence in circulation that may induce systemic anomalies associated with PE. Moreover, *cis* P-tau also significantly induced the production of sFlt-1 and sEng (Fig. 3g), anti-angiogenic factors associated with the PE etiology.

### Robust detection of *cis* P-tau in human PES

To independently confirm the presence of *cis* P-tau in PES, we used our unique blood test for detection of proteinopathy markers for PE and AD^46^. The basic premise of this test is that protein aggregates accumulate with time in autophagy-deficient trophoblasts as these cells fail to degrade large complexes of protein aggregates^55,56^, which can be detected by ProteoStat^46^, an immunofluorescence rotor dye that shows unique affinity for aggregated proteins^56^.

**Fig. 4:**
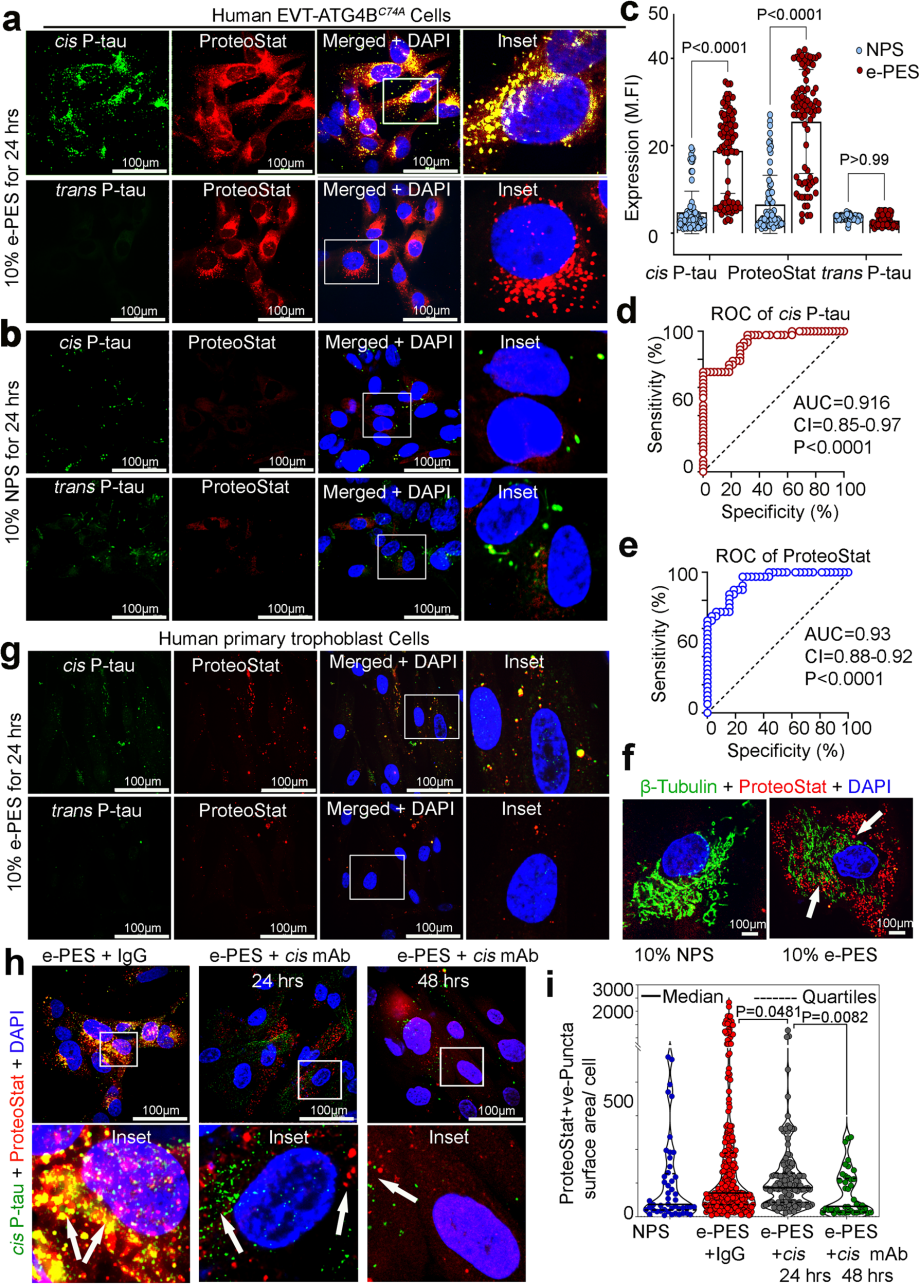
Robust detection of *cis* P-tau in human PES. Autophagy-deficient trophoblast cells (ADTs) were incubated for 24 h with 10% of PES (e-PES; *n* = 34) (**a**) or NPS (*n* = 28) (**b**), followed by immune-colocalization with *cis* P-tau (green) and ProteoStat (red) or *trans* P-tau (green) and ProteoStat (red). Representative merged and inset confocal images are depicted showing colocalization. Scale bar: 100 μm. **c** Mean fluorescence intensity (MFI) quantification of *cis* P-tau, *trans* P-tau and proteo-Stat positive protein aggregates from the experiment in **a** and **b** were shown. PES, *n* = 34; control, NPS, *n* = 28, 2-3 coverslips were analyzed per donor, two-way ANOVA followed by Bonferroni’s post hoc test; mean ± s.e.m. **d** ROC curve analysis of *cis* P-tau abundance for prediction of PE (*n* = 38) and NP (*n* = 38). Area under the ROC curve (AUC) = 0.9162, std error = 0.03043, 95% confidence interval (CI) 0.8566 to 0.9759. *P* value = <0.0001. **e** ROC curve analysis of ProteoStat abundance for prediction of PE (*n* = 38) and NP (*n* = 38). Area under the ROC curve (AUC) = 0.9346, std error = 0.02786, 95% confidence interval (CI) 0.8800 to 0.9892. *P* value = <0.0001. **f** PES disrupts the β-tubulin positive microtubule network in ADT cells. Clusters of ProteoStat positive protein aggregates were found near disrupted microtubule networks (arrowhead). *n* = 5. Bar: 100 μm. **g** There was no substantial accumulation of *cis* P-tau and protein aggregates when autophagy-proficient human primary trophoblasts were treated with NPS or PES. Bar: 50μm. *n* = 5. **h** *Cis* mAb blocks and disaggregates PES-induced *cis* P-tau induction and aggregation. ADT cells were treated with 10% PES or NPS for 24 h before being incubated with *cis* mAb or IgG isotype for a further 24 and 48 h, followed by staining with *cis* P-tau and ProteoStat. **i** A violin plot illustrating the frequency, distribution, and area of ProteoStat positive protein aggregate puncta after neutralization with IgG and *cis* mAbs. Solid and dashed black lines represent the median, upper and lower dashed black line represents 3rd quartiles (75%) and 1st quartiles (25%) and the quartiles of the distributions, respectively. Statistics are represented in the figure derived from one way ANOVA followed by Tukey’s post hoc test; mean ± s.e.m. Puncta area was analyzed by ImageJ from NPS (*n* = 17), e-PES+ IgG isotype (*n* = 15), e-PES+ *cis* mAb 24 h (*n* = 20), e-PES+ *cis* mAb 48 h (*n* = 19) cells in each condition.

Genetically engineered autophagy-deficient human EVT-ATG4B^C74A^ trophoblast cells^55,56^ and primary human trophoblasts were exposed to 10% (v/v) PES or NPS samples for 24 h. Cells were then fixed and stained for *cis* P-tau or *trans* P-tau and ProteoStat to confirm the aggregated nature of these proteins. Robust signal of ProteoStat^+^ aggregated *cis* P-tau, not *trans* P-tau, was detected in EVT-ATG4B^C74A^ cells exposed to PES, but not NPS (Fig. 4a, b). This method was used to analyze a larger serum sample size from PE (*n* = 38) and normal pregnancy (*n* = 38) and correlated with ProteoStat staining^54^. *cis* P-tau was mainly detected in PES samples compared to NPS controls (Fig. 4c). The ROC curve analysis for the ProteoStat staining parallels that of *cis* P-tau (Fig. 4d, e), whereas this analysis did not reach sensitivity and specificity relationship for *trans* P-tau (Supplementary Fig. 14a). These results provide strong evidence of sensitivity and specificity of the blood *cis* P-tau assay as a PE biomarker. Of note, in autophagy-proficient primary human trophoblasts, no *cis* P-tau accumulation could be detected when incubated with PES under similar conditions for 24 h (Fig. 4g, Supplementary Fig. 14b), as expected. However, these primary human trophoblasts could accumulate *cis* P-tau aggregates when incubated with PES, but not NPS for 72 h (Supplementary Fig. 14c, d, e), suggesting PES-induced disruption of autophagy activity in these cells after longer incubation. Importantly, PES also disrupted β-tubulin organization, which appeared to be normal in NPS-treated trophoblasts (Fig. 4f). Notably, when EVT-ATG4B^C74A^ cells were incubated with PES for 24 h, *cis* P-tau robustly accumulated in these cells. However, *cis* P-tau mAb, but not control IgG treatment for 24 h or 48 h significantly blocked accumulation of *cis* P-tau aggregates (Fig. 4h, i). Immunoblot analysis also confirmed the presence of *cis* P-tau in PES, but not NPS samples (Supplementary Fig. 14f). Thus, *cis* P-tau is robustly detected in PES.

### Depletion of *cis* P-tau from PES prevents their ability to cause the PE-like pathological and clinical features in pregnant humanized tau mice

Given robust *cis* P-tau in PES, the critical question is whether such *cis* P-tau contributes to the development of any pathological or clinical features in PE. In this regard, we have previously shown that single i.p. injection of 100 µl of human PES on gestational day (gd) 10 is sufficient to induce robust PE-like features in pregnant IL-10^−/−^ mice, including classical clinical features of elevated blood pressure, proteinuria, glomerular endotheliosis, and fetal and placental growth restriction^81^. To evaluate the significance of *cis* P-tau in the PE pathophysiology and efficacy of its mAb in treating PE, we used humanized tau mice for serum injection as *cis* P-tau is induced at a very low level in these humanized tau mice at 2-3 months and might provide a substrate for PES *cis* P-tau-mediated seeding^40^ as needed for in vivo protein aggregation. Moreover, the seeding capability of AD-derived CSF tau has been demonstrated in these humanized mice^82^.

**Fig. 5:**
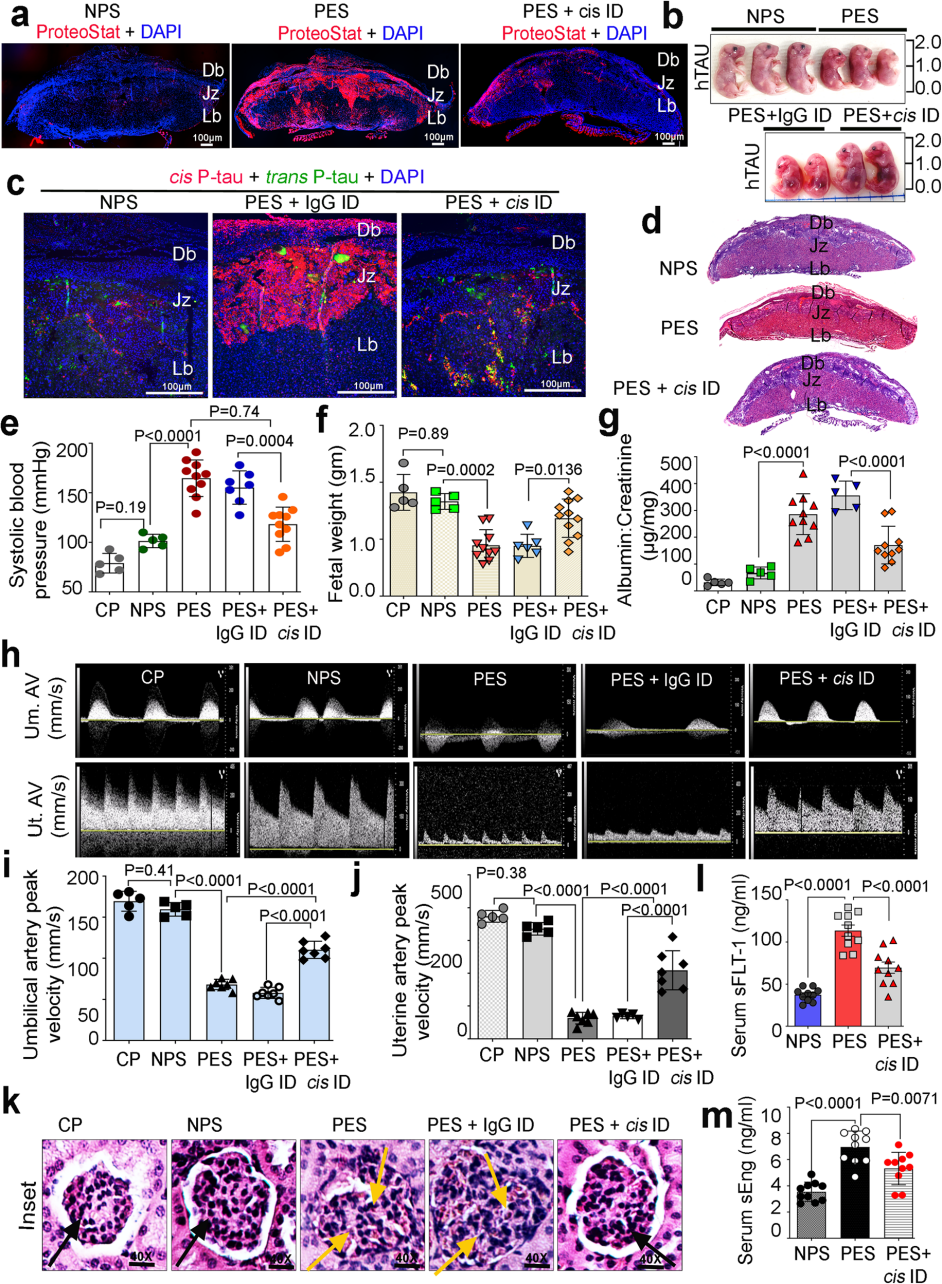
*Cis* P-tau immunodepletion from PES effectively prevents the development of PE-like pathological and clinical features in humanized tau mouse model of PE. Pregnant htau mice were injected (I.P) on gestational day 10 (gd10) with 100 µl saline (control; *n* = 5), NPS (*n* = 5), PES (*n* = 10), IgG isotype immunodepleted PES (PES + IgG ID; *n* = 7), or *cis* mAb immunodepleted PES (PES+ *cis* ID; *n* = 10) before being subject to the following assays. **a** Confocal images of placental slices on gd 17.5 reveal ProteoStat positive protein aggregation formation after PES induced PE, which was reversed by *cis* P-tau depletion. Scale bars, 1 mm. Db = Maternal decidua basalis, Jz = Junctional zone, Lz = Labyrinth zone, **b** Representative gross images of fetuses on gd 17.5 exhibit PES-induced intrauterine growth restriction, which were reversed by *cis* P-tau depletion. **c** Representative confocal images of placentas from NPS, + IgG ID and PES + *cis* ID-treated htau mice reveal PES-induced strongest placental expression of *cis* P-tau in the junctional zone and decidia basalis, which was reversed by *cis* P-tau depletion. *n* = 5–7 placental sections. **d** Microscopic morphologies of the placenta on gd 17.5 reveal PES-induced placental histology, which was revered by *cis* P-tau depletion. Three layers of decidua basalis (Db), junctional zone (Jz) and labyrinth (Lb) are indicated. H&E staining, 20× magnification. **e** The systolic blood pressure measurements of mice on gd17.5 reveal PES-induced systolic hypertension, which was reversed by *cis* P-tau depletion. **f** Weight measurements of individual embryos on GD17.5 reveal PES-induced intrauterine growth restriction, which was reversed by *cis* P-tau depletion. **g** Spot urine albumin/creatinine ratio evaluations on gd17.5 reveal PES-induced proteinuria, which was reversed by *cis* P-tau depletion. Representative pulse-wave Doppler ultrasound images (**h**) and analyses of systolic velocities (**i**, **j**) of umbilical artery and uterine artery on GD16.5 reveal PES-induced decreases in umbilical and uterine artery systolic velocities, which were reversed by *cis* P-tau depletion. **k** Representative images of H&E-stained histopathological analysis of sagittal kidney sections reveal PES-induced glomerular endotheliosis, which was reversed by *cis* P-tau depletion. black arrow, normal glomeruli; yellow arrow, glomerular endotheliosis. Measurements of serum sFLT-1 (**l**) and sEng (**m**) concentrations on gd17 reveal PES-induced elevation of sFLT-1 and sEng, which was reversed by *cis* P-tau depletion. Data are presented as mean ± s.e.m. and analyzed by two-way ANOVA followed by Bonferroni post-tests, *n* = 10.

PES was used as such or immunodepleted for 72 h with *cis* mAb or IgG isotype. Efficient immunodepletion (ID) was confirmed by incubating autophagy-deficient EVT-ATG4B^C74A^ cells with *cis* P-tau immunodepleted PES and by evaluating the presence of *cis* P-tau aggregates (Supplementary Fig. 15a). *Cis* P-tau depleted PES samples were then tested for their inability to induce PE-like pathology and symptoms in pregnant humanized tau mice. Pregnant mice (Supplementary Fig. 15b for mating strains) were injected i.p. on gd 10 with no serum (*n* = 5), normal pregnancy serum (NPS, *n* = 5), preeclampsia serum (PES, *n* = 10), PES depleted with IgG (*n* = 7), and PES depleted with *cis* Ab (*n* = 10). First, we evaluated whether PES injection in pregnant mice induced accumulation of protein aggregates in the placenta. Indeed, robust ProteoStat-positive protein aggregation signals were detected in the placenta, particularly in the junctional zone (Fig. 5a), which could be inhibited by using *cis* P-tau-depleted PES (Fig. 5a). PES-induced protein aggregation in the placenta was associated with fetal growth restriction (Fig. 5b). These results are consistent with the junctional zone propensity of protein aggregate accumulation in the placenta-specific conditional knockout of the autophagy ATG7 gene^83^. Importantly, *cis* P-tau depletion by its mAb blocked accumulation of its protein aggregates in the placenta (Fig. 5c, and Supplementary Fig. 15c). Exposure of pregnant mice to PES, but not NPS, also led to the significant decrease in the placental weight and the litter number, both of which were rescued by depletion of *cis* P-tau using its mAb, but not control IgG (Supplementary Fig. 15d, e).

Pregnant mice were subjected to urine collection on gd 16, blood pressure measurement on gd 17, and collection of serum and placental and fetal units for further analysis of weight. *Cis* P-tau depletion by its mAb, but not control IgG, normalized placental morphology (Fig. 5d). Importantly, PES-induced elevated blood pressure, proteinuria, and fetal weight were normalized by *cis* P-tau depletion (Fig. 5e–g). We also measured umbilical and uterine arterial systolic velocities using Pulse-wave Doppler Ultrasound imaging recorded on gd 16.5. Whereas NPS or no serum showed little or no effects, PES had adverse effects on umbilical arterial and uterine arterial velocity, which were largely rescued by depletion of *cis* P-tau by its mAb, but not control IgG (Fig. 5h–j). Moreover, PES also induced the PE-like characteristic glomerular lesion, which was also rescued by depletion of *cis* P-tau by its mAb, but not control IgG (Fig. 5k, and Supplementary Fig. 15f), indicating the effects are highly specific to *cis* P-tau. As previously described^81^, PES also induced elevated production on sFlt-1 and sEng which were also normalized by *cis* P-tau depletion (Fig. 5l, m). Thus, elimination of *cis* P-tau from PES using stereo-specific mAb not only restores the crosstalk between extravillous trophoblasts and endothelial cells in vitro, but also prevents the onset of PE-like pathological and clinical features in pregnant humanized mice.

## Discussion

Here we discover the significance of *cis* P-tau across a spectrum of PE-associated pathological and clinical outcomes using an array of complementary in vitro, in vivo, and ex vivo approaches. We find robust *cis* P-tau in the placenta and blood of early- or late-onset PE patients, as well as in primary human trophoblasts exposed to hypoxia or PE patient sera due to Pin1 inactivation. To delineate the pathogenic role of *cis* P-tau in the onset of PE-like features, we discover that humanized tau mice with the capacity of tau seeding could be successfully used to recapitulate all the classical features of PE in response to sera from PE patients. Importantly, depletion of *cis* P-tau from the PE patient sera protects against the onset of all the PE-like pathological and clinical features. Thus, blood *cis* P-tau is a central early driver and a promising biomarker of PE and can be effectively targeted by stereospecific mAb for early diagnosis and treatment. Our results represent the first example where *cis* P-tau drives disease development outside of the brain and in a younger population represented by pregnant women. As *cis* P-tau is an early disease driver and blood biomarker in incipient AD patients and *cis* P-tau mAb is in clinical trials, these results not only discover a previously unknown early pathogenic mechanism in PE and its causal molecular link with dementia, but also offer an early biomarker and effective immunotherapy for PE.

*Cis* P-tau is an early blood biomarker and disease driver in patients with incipient AD, TBI/CTE or stroke/VaD^31,37,39,40,44,46^, but nothing is known about the role of tau in PE. Tau immunotherapy in AD, TBI, CTE, and other neurodevelopmental disorders enhances neuronal functions by eliminating or depleting harmful proteins^31–33,39,40,84,85^. Based on the results presented here on *cis* P-tau and other phosphorylated tau proteins in the PE placenta, we suggest that PE shares the tauopathy etiology with AD, TBI/CTE, stroke/VaD, and possibly other neurological disorders. We have used the stereo-specific *cis* P-tau mAb to demonstrate the presence of this toxic protein in the PE placenta and serum and to specifically inhibit its pathogenic activity in clinically relevant in vitro, in vivo, and ex vivo models of PE. We have found for the first time robust *cis* P-tau in the placenta and sera from PE patients and hypoxia-exposed primary human trophoblasts. We have also provided first evidence in the non-neuronal tissue that Pin1 is inactivated by oxidation and phosphorylation, and associated with *cis* P-tau conformational transition in the PE placenta. Although *Pin1* knockout has been used to demonstrate its role in tau conformational change from *trans* P-tau to *cis* P-tau^28–31^, our results provide evidence for disease-associated inactivation of Pin1 and concurrent onset of pathogenic *cis* P-tau isoform in PE. Notably in AD, TBI/CTE and stroke/VaD, Pin1 is also catalytically inactivated by Cys113 oxidization^38,39^ or Ser71 phosphorylation by DAPK1^40,41,68^. Our results support that DAPK1 activation also contributes to Pin1 inactivation in PE. Moreover, blood *cis* P-tau can be detected early in pregnancy, making it a potential early biomarker for PE. Notably, blood *cis* P-tau is an early biomarker for human patients with incipient AD^44^ and pre-clinical AD^45^, as well as after TBI, with a 10- to 15-fold increase within 24 h after severe TBI^46^. Although more clinical samples are needed to define its diagnostic and prognostic value, *cis* P-tau may be a promising early blood biomarker for PE.

Proteopathic seeds, upon intercellular transmission^29,86,87^, might potentiate protein aggregation in unaffected cells in two ways. First, it decreases the lag phase of protein misfolding and recruits monomeric proteins to form aggregates^88^. Our findings suggest that once *cis* P-tau reached a threshold amount intracellularly, it could start forming aggregates which could be released into the circulation. Most importantly, trophoblast endovascular invasion of the decidua and spiral artery remodeling are compromised in PE. Our results suggest that *cis* P-tau-mediated toxic effects in the placenta may impede trophoblast invasion and endovascular activity during early placentation^79^. Moreover, the production of anti-angiogenic factors such as sFlt-1 and sEng detected in PE patients was induced in hypoxia-exposed primary human trophoblasts. Interestingly, PE-serum induced trophoblast invasion deficiency, endovascular activity defects, and production of anti-angiogenic factors could be rescued by *cis* P-tau depletion by stereo-specific mAb in hypoxia-exposed or PES-treated human trophoblasts. In addition, our results provide intriguing evidence that tau phosphorylation and aggregation together with microtubule disassembly directly correlate with trophoblasts’ antiangiogenic outcomes. We have previously shown that early onset PE placenta is impacted by sterile inflammation/pyroptosis^66^. We have published that pyroptosis is induced in autophagy-deficient human trophoblasts treated with sera from PE patients as well as in primary human trophoblasts exposed to hypoxia^65^. Exposure to hypoxia elicits excessive ER stress-mediated unfolded protein response (UPR) and activation of NOD-like receptor pyrin-containing (NLRP3) inflammasome in primary human trophoblasts^65^. Our current attempts are focused on examining whether these inflammatory triggers induce Pin1 inactivation and *cis* P-tau conformational regulation. Since pathologies such as neurodegeneration may be associated with non-infectious inflammation^89,90^, such information will be very useful overall. The presence of robust levels of toxic tau aggregates in the trophoblast layer may interfere with cytotrophoblast proliferation and fusion into syncytiotrophoblasts (STBs)^91^. Since STBs are not able to replicate, villous cytotrophoblasts (CTBs) fuse with overlying STBs to bring fresh cellular components to STBs for their homeostasis. This CTB-STB exchange may be adversely affected by placental *cis* P-tau and phosphorylated tau aggregation^92,93^.

We have provided first direct evidence that *cis* P-tau present in serum is sufficient to induce all severe PE-like pathological and clinical features in pregnant humanized tau mice. Importantly, these PE-like features were largely mitigated by elimination of *cis* P-tau from PES prior to administration to the pregnant mice. PES caused the PE features including accumulation of protein aggregates in the junctional zone, elevated blood pressure, proteinuria, fetal growth restriction, and glomerular endotheliosis^83^. *Cis* P-tau depletion corrected all these features and restored normal pregnancy. Interestingly, in vivo evaluation by high resolution ultrasound device of umbilical and uterine arterial systolic velocities confirmed these results. Similar to normal pregnancy serum, *cis* P-tau depleted PES did not cause any significant anomalies in these readings. The therapeutic impact of *cis* P-tau elimination by its mAb has been demonstrated in the settings of AD, TBI/CTE and stroke/VaD^31,39,40^. These results indicate that the Pin1-*cis* P-tau axis not only is a common stress responsive mechanism, but also offers promising diagnostics and therapeutics using stereo-specific mAb.

In summary, we demonstrate for the first time that blood *cis* P-tau, which is likely released from stressed placenta due to Pin1 inhibition, is a central etiologic driver and biomarker of PE, and that such disease driver can be effectively neutralized by stereo-specific mAb for treating PE. As *cis* P-tau is an early driver and biomarker for incipient and preclinical AD, stroke/VaD, TBI/CTE and *cis* P-tau mAb is in clinical trials, our results not only discover an early disease driver, but also offer an early biomarker and effective antibody therapy for PE.

## Methods

### Human subjects

The guidelines from the Task Force on Hypertension in Pregnancy were strictly followed for the diagnosis of PE patients. According to the gestational age at the time of diagnosis and/or delivery, PE is mechanically divided into two types: early-onset (e-PE) (pregnancy ending before 34 weeks) and late-onset (l-PE). The ACOG definition was used to define early onset and late onset PE patients. For human PE serum samples, all control serum samples came from gestational age-matched healthy (normal) pregnancies and correlated with gestational age of early onset or late onset phenotype of preeclampsia. For control placental tissue, it is not possible to collect placental tissue from normal pregnancy during weeks 28–34. These control placental tissue samples were derived from preterm birth deliveries during this gestational age period. Preterm birth placental tissues were not found to have any evidence of protein aggregates, again supporting the unique concept of proteinopathy in early or late onset preeclampsia. For late onset preeclampsia placental samples, control placental tissue was derived from normal term deliveries. Supplementary Figs. 2b and 3b tables include the demographics of patients who have been enrolled for collection placental tissue and serum samples. Exclusion criteria included chronic hypertension, gestational or pre-existing diabetes, fetal demise, daily cigarette use, fetal abnormalities, and multiple pregnancies for the study. Serum was isolated within 30 min of each participant’s blood being obtained in 7–9 ml BD Vacutainer SSTTM tubes. After aliquoting the serum in small amounts, the samples were stored at −80 °C until further usage. All blood samples were taken prior to steroid administration for fetal lung maturity in instances with pregnancies 34 weeks gestation (either PE patients or gestational age matched controls) in these situations. A 1-cm^3^ piece of the placenta was extracted and vigorously washed with cooled phosphate-buffered saline solution for the collection of placental samples. After extracting any remaining blood from placental tissue, a portion was kept at −80 °C for future use, while the remainder was fixed in 10% formalin for immunohistochemistry analysis. All protocols concerning the use of human material were approved by the Institutional review board of Women and Infants Hospital. All subjects gave their informed consent prior to participating in the study.

### Mice

All animal protocols were approved by the Lifespan Institutional Animal Care and Use Committee. Female transgenic humanized tau (htau) mice expressing human Tau and lacking the endogenous mouse Tau gene (Homozygous for Mapttm1(EGFP)Klt, Hemizygous for Tg(MAPT)8cPdav) were mated with non-carrier male (Homozygous for Mapttm1(EGFP)Klt > , Non Carrier for Tg(MAPT)8cPdav)^40,94,95^. All mice were allowed free access to food, water, and activity. Mice used were 8–10 weeks of age at the time of mating and PE-like features observed as described^81^. The day of vaginal plug appearance was designated gestational day (gd) 0. Sulfopin injected i.p. once daily for seven days with 20, 10, 5 mg/kg from gd9-gd15 to evaluate its effectiveness during pregnancy. After evaluation, 5 mg/kg Sulfopin was injected i.p. once every other day between gd9-gd15. The formulation was 5% N-methylpyrrolidone, 5% solutol and 20% DMSO. Pregnant htau mice were injected i.p. serum samples (100 μl) from e-PE (*n* = 11) or l-PE (*n* = 7) patients on gd 10 or an equivalent amount (100 μl) of normal pregnancy serum (*n* = 9). Specific antibodies to *cis* P-Tau*, trans* P-tau, or isotype control IgG were used to immune-deplete serum of respective phosphorylated tau proteins. Total urinary albumin was measured using Albumin (mouse) ELISA kit (ALPCO Diagnostics, Germany). To normalize the albumin, urinary creatinine was measured using Metra Creatinine Kit (Quidel Corporation, San Diego, CA), according to the manufacturer’s protocol. Proteinuria was represented as the ratio of urinary albumin/creatinine. On gd17, blood pressure was recorded by the tail-cuff method as described earlier^81^. The animals were then euthanized, and fetal weights were recorded. The kidneys and placentas were fixed in 10% formalin and paraffin-embedded for section. Sections from the kidney and placenta were stained with H&E for histopathological examination as previously described^52,81^.

Doppler ultrasonography was used to evaluate uterine and umbilical artery blood flow on gd 16. (Vevo3100, VisualSonics, Canada)^96–98^. These measurements included at least three cardiac cycles that were not impacted by maternal movements. The peak systolic and diastolic velocities of the uterine and umbilical arteries were determined in order to evaluate changes in blood flow using the software used to analyze the data (Vevo LAB 3.1.1, VisualSonics, Canada).

### Antibodies

Specific antibodies to *cis* P –tau, *trans* P-tau, and unique peptidyl-prolyl *cis*-*trans* isomerase, *Pin1* were generated as described earlier^37–39,68^. Other antibodies were purchased from commercial sources. A list of the antibodies used and their specific applications is provided in the supplementary methods.

### Chemical inhibitors/inducers

The IRE1 RNase inhibitor 4μ8c was purchased from EMD Millipore (catalog no. 412512), ER Stress Inducer Tunicamycin was purchased from Tocris (catalog no. 3516), potent and selective, ATP-competitive inhibitor of death-associated protein kinase 1 (DAPK1) TC-DAPK 6 (catalog no. 4301). Pin1 inhibitor Sulfopin (PIN1-3), which is a highly selective covalent inhibitor of Pin1was purchased from Selleckchem (Catalog No.S9782).

### Immunodepletion of *cis* P-tau in serum samples from preeclampsia patients (PES)

For the pre-cleaning of the preeclampsia serum (PES), protein G-Sepharose 4 Fast Flow beads (GE Healthcare) were first washed three times in ice-cold PBS, pH 7.4^99,100^. The serum samples were then mixed with the pre-washed beads and incubated on a rocking platform overnight at 4 °C, after which the mixture was centrifuged at 600 × g for 5 min, and the supernatant was transferred to a new tube that was labeled “input1.”To prepare the antibody-bound beads, an appropriate amount (20 μg) of antibodies (*cis* mAb, *trans* mAb and IgG) were incubated together with 120 μl of washed beads in IP binding buffer (PBS, pH 5.5) and incubated on a rocking platform overnight at 4 °C. The mixture was then centrifuged at 600 × g for 5 min, followed by three washes of the antibody-bound beads in ice-cold IP buffer. To immunodeplete the protein complexes, 1 mg of the pre-cleaned “input” was added to the antibody-bound beads and incubated on a rocking platform 72 h at 4 °C. After centrifugation for 5 min at 600 × g at 4 °C, the supernatant was carefully removed and saved as “immune depleted” (ID). The beads were washed extensively (by turning the tube upside down 10 times) with IP washing buffer to obtain complexes of proteins bound to the antibody-coated beads (saved as IP).

### Western blotting

For western blotting, cells were lysed in 1× lysis buffer (Roche) supplemented with phosphatase and protease inhibitors (1 µg/ml aprotinin, 1 µg/ml pepstatin, 1 µg/ml leupeptin, 1 mM phenylmethane sulfonyl fluoride, and 1 µg/ml trypsin inhibitor) and 1% Triton X-100, followed by sonication and incubation on ice for 2 h. Subsequently, the cells were centrifuged at 7500 rpm for 30 min at 4 °C. The supernatants were collected, and protein quantities were estimated by the Bradford method; 25–50-μg total protein aliquots from each sample were resolved by 10–12% SDS-PAGE.

### Lambda phosphatase treatment

To establish the phosphorylation status of tau proteins in the human placenta, we incubated placental protein extracts (120 µg) from the PE and gestational age-matched control pregnancy for 1.5 h with 1200 units of lambda phosphatase (λ PPase) (New England BioLabs Cat# P0753S)^101^. Samples were immediately put on ice and ready for SDS-PAGE analysis after incubation. A 4–12% Bis-Tris polyacrylamide gel was loaded with lysates (20 ug) and run at 120 volts for 10 min. Proteins were transferred to PVDF membrane and incubated with anti-Tau phospho T231 (pT231,1:2000), phospho Serine 396 (p396 p-Tau, 1:500), *cis* P-tau and *trans* P-tau overnight at 4 °C. Goat anti-Rabbit IgG (H + L), HRP (1:2000; ThermoFisher Catalog # 32460) and Rabbit anti-Mouse IgG (H + L), and HRP (1:2000; ThermoFisher Catalog # PA1-28568) secondary antibodies were used to detect tau phosphorylation status.

### Co-immunoprecipitation (Co-IP)

The placenta was homogenized in 10 volumes of TBS with a protease inhibitor cocktail and a phosphatase inhibitor cocktail (50 mM Tris-HCl pH 8.0, 274 mM NaCl, and 5 mM KCl). The homogenates were centrifuged at 25,000 × g for 25 min at 4 °C.The pellets were lysed in a non-denaturing lysis buffer containing 1% protease and 1% phosphatase inhibitor for 20 min before being centrifuged at 12,000 rpm (4 °C). Four micrograms of Pin1 (Cell signaling, cat#3722) or IgG (Thermo fisher, cat#31235) were pre-incubated for 8 h at RT with DynabeadsTM Protein G (Thermo fisher) and washed five times with DynabeadsTM Protein G Immunoprecipitation Kit buffer (Thermo fisher,10007D). The extracted protein was then treated for 24 h at 4 °C with a primary antibody linked with DynabeadsTM Protein G beads. The protein-beads combination was rinsed five times with kit buffer before being boiled for 5 min and centrifuged at 10,000 rpm for 5 min to obtain the supernatant. The bound proteins were then examined using a western blot.

### The identification of protein aggregates within the placenta and trophoblast cells

Aggregated proteins were detected using ProteoStat Aggresome Detection Kit (ENZO, ENZ-51,035-K100) according to a modified protocol described previously^56,102^. Briefly, fixed human placental sections were de-paraffinized and then treated with 0.5% Sudan Black B for 20 min at room temperature. Sections were then fixed and washed in PBS for 15 min at 37 °C. Tissue sections were stained with ProteoStat dye for 15 min at room temperature. Nuclei were stained with DAPI (Vector laboratories, H-1200). The intensity of ProteoStat specific signal in each placental section was measured from at least 35 villi from 10 randomly selected fields at ×40 magnification using Nikon A1R software. For detection of protein aggregates in trophoblast cells, autophagy-deficient trophoblasts (ADT) cells (ATG4B^C74A^) or primary trophoblasts cells were used recently described by us^56,103^. All images were acquired with a 60 × objective lens. The signal intensity was measured using ImageJ software (NIH). Figures were processed with brightness/contrast adjustment using Photoshop CS3 (Adobe) using the same settings.

### Isolation of placental soluble and insoluble fractions for localization of *cis* P-tau

Detergent Sarkosyl soluble and insoluble fractions were extracted from frozen placental tissues. To obtain the Sarkosyl-insoluble fraction, we followed the steps outlined previously^104,105^. In brief, the placenta was homogenized in 10 volumes of TBS (50 mM Tris-HCl pH 8.0, 274 mM NaCl, and 5 mM KCl) containing a protease inhibitor cocktail and a phosphatase inhibitor cocktail. The homogenates were centrifuged for 25 min at 4 °C at 25,000 × g. Fraction A was formed from the resultant supernatants. The pellets (B) were re-homogenized in 5x volumes of high salt and sucrose buffer including protease inhibitor cocktail and phosphatase inhibitor cocktail (10 mM Tris-HCl, pH 7.4, 0.8 M NaCl, 10% sucrose, 1 mM EGTA) and centrifuged at 25,000 × g for 25 min at 4 °C. The supernatants (fraction C) were then incubated for 1 h at 37 °C with 1 percent Sarkosyl (Sigma) before being centrifuged at 150,000 g for 1 h at 4 °C. In TE buffer, the pellets (D) were suspended. The fraction C was employed as the Sarkosyl soluble fraction, whereas fractions A + D were used as the insoluble fractions.

### Tau immunoprecipitation

Sarkosyl soluble and insoluble fraction was pre-cleaned for immunoprecipitation using Dynabeads Protein G (Life Technologies). The supernatants were then mixed with the pre-washed beads and incubated on a rocking platform overnight at 4 °C, after which the mixture was centrifuged at 600 × g for 5 min, and the supernatant was transferred to a new tube that was labeled “input.” To immunoprecipitate the protein complexes, 1 mg of the pre-cleaned supernatants was added to the antibody-bound beads and incubated on a rocking platform overnight at 4 °C. After centrifugation for 5 min at 600 × g at 4 °C, the supernatant was carefully removed and saved as “flow-through” (FT). The beads were washed extensively (by turning the tube upside down 10 times) with an IP washing buffer to obtain complexes of proteins bound to the antibody-coated beads (saved as IP). The set of samples destined for Western blot analysis was directly boiled in 1× SDS-PAGE sample buffer (50 mm Tris-HCl, pH 6.8, 2% SDS, 10% glycerol, 1% β-mercaptoethanol, 12.5 mm EDTA, and 0.02% bromphenol blue) (24), followed by SDS-PAGE and Western blot analysis.

### In vitro hypoxia and reoxygenation (H/R) treatment

The experimental design for H/R treatment of primary human trophoblasts (PHTs) followed our previously described method^55,66^. Human primary trophoblasts were isolated from the placenta at 19 weeks of gestation (purchased from ScienCell Research Laboratories) and cultured as described by the vendor. Primary human trophoblasts (PHTs) were initially seeded at a density of 300,000 cells/cm2 and cultured in Dulbecco’s Modified Eagle Medium (DMEM) (ThermoFisher Cat# 10569010) for a period of 4 h. The culture conditions during this incubation period were specifically designed to promote the differentiation of the PHTs into syncytiotrophoblasts. Following the 4-hour incubation phase, the PHTs were carefully transferred to a hermetically sealed incubator (Thermo Electron in Marietta, OH, USA). This specialized incubator was equipped with a highly sensitive sensor that was connected to a data acquisition module provided by Scope (Marlboro, MA, USA). One group of PHTs was exposed to normoxia while another group was subjected to low oxygen tension (1%). These varying oxygen conditions were maintained for a total duration of seventy-two hours. Before introducing the culture media to the PHTs, the gas mixture within the hermetically sealed incubator was carefully pre-equilibrated to ensure the specific oxygen concentration required for each experimental group. Throughout the entire duration of the experiment, the culture media was renewed every 24 h to provide the PHTs with fresh nutrients and maintain a stable environment conducive to their growth and differentiation. Following hypoxia, the cells were incubated for 3 h at 37 °C in a humidified 5% CO_2_ atmosphere, to accelerate the return towards the normoxic 16% O_2_ phase. As control, cells were also exposed to normoxic conditions in each experiment.

### Generation of autophagy-deficient trophoblasts

An autophagy-deficient (AD) cell line (ATG4B^C74A^) was generated by transfecting immortalized extravillous trophoblasts HchEpC1b cell line with vector containing ATG4B dominant negative mutation at C74A as described previously^55,106^. Briefly, HchEpC1b-ATG4B^C74A^ cells (ATG4B^C74A^), was constructed by stable transfection with pMRX-IRES-puromStrawberry-ATG4B^C74A^, containing a point mutation in ATG4B at C74A that inhibits MAP1LC3B-II formation, resulting in defective autophagosome closure. Control HchEpC1b cells were stably transfected with pMRXIRES- puro-mStrawberry, a control vector only encoding monomeric red fluorescent protein35. Cells were routinely cultured in RPMI1640 medium (GIBCO, 11875, USA) supplemented with 10% FBS (GIBCO, 15140) 100 U/ml penicillin and 100 μg/ml streptomycin (GIBCO,15140) at 37 °C in a 5% CO2 atmosphere.

### Antibody-mediated intracellular neutralization assay

Hypoxia or serum-treated trophoblast cultures were incubated with *cis* P-tau, or *trans* P-tau specific mAbs or mouse IgG isotype at a concentration of 100 ng/ml as determined by total human tau ELISA (Thermofisher KHB0041). Cells were fixed for immunofluorescence or lysed (after 24 h following the addition of mAbs as described earlier^39,40,43^. Prior to fixation or protein separation, the treatment media (conditioned medium; CM) was removed and cells were washed twice in PBS to remove extracellular tau. Experiments were always carried out in serum free-media, unless otherwise noted. Except where noted, all experiments were conducted in triplicates with *n* = 3 wells per treatment, *n* = 9 total.

### Lactate Dehydrogenase (LDH) release assay

The release of lactate dehydrogenase into the extracellular space/supernatant is thought to be a key characteristic of plasma membrane integrity breakdown. The LDH Assay Kit (Cytotoxicity) (ab65393) was used for in vitro investigation to assess LDH produced from necrotic cells in various groups. In summary, centrifuge cells at 600 × g for 10 min to precipitate the cells, and then cell-free culture supernatants were collected from each well of the plate and incubated with the working reagent mixture for 30 min at room temperature, as directed by the manufacturer. At 490 or 450 nm, the optical density of each well in the assay, which is proportional to the LDH activity and percentage of necrotic cells, was measured using a microplate reader. The wavelength of reference should be 650 nm. Ten independent tests were used to calculate the percentage of necrotic cell death by measuring the optical density of the treated group minus the control group/LDH releasing, reagent treated group (high control) minus the control group.

### Collection and concentration of trophoblast conditioned media (CM) under hypoxia –reoxygenation

The following steps were taken to create a conditioned media: PHTs were grown under H/R conditions for 72 h, and then incubated with *cis* mAb, *trans* mAb, and IgG antibodies for an additional 24 h as described earlier. All the conditioned media (CM) from each 6 well plate was aspirated after incubation. For the removal of cell debris, CM samples were centrifuged at 3000 rpm for 10 min and then filtered through a 0.22 μm membrane filter. Centrifugal filter units with a 3 kDa cut-off (Millipore, Billerica, MA) were used to concentrate the medium by a factor of 50 using the manufacturer’s instructions to make 300 μl per 1×10^5^ cells. We utilized all the concentrated trophoblast for the Transwell migration experiment or stored it at −80 °C until use.

### Real-time quantitative PCR (RT-PCR)

Total RNA was isolated using RNeasy mini kit (Qiagen) according to the manufacturer’s instructions, and concentrations were determined using a NanoDrop 1000 spectrophotometer (NanoDrop Technologies). Using the First Strand cDNA synthesis kit (Invitrogen), one microgram of RNA was reverse-transcribed into cDNA. RT-PCR was used to measure mRNA expression using the Applied Biosystems™ 7500 Real-Time PCR and SYBR GreenER™ qPCR SuperMix Universal (Invitrogen) The primers included sFlt-1i13 forward: ACAATCAGAGGTGAGCACTGCAA, sFlt-1i13 reverse: TCCGAGCCTGAAAGTTAGCAA, sFlt-1e15a forward: ACACAGTGGCCATCAGCAGTT, sFlt-1e15a reverse: CCCGGCCATTTGTTATTGTTA, Endoglin Forward: AGGCGGTGGTCAATATCC, Endoglin Reverse: AAGTGTGGGCTGAGGTAG, 18 S forward: TAACGAACGAGACTCTGGCAT, and 18 S reverse: CGGACATCTAAGGGCATCACAG. 18 S was used as a reference house-keeping gene. The relative changes in gene expression levels between the treated and control samples were determined by dividing the treated samples’ normalized values by the control samples’ normalized values.

### Transwell migration assay

For the trophoblast cell migration test, fresh PHTs were adjusted to a density of 2.5 × 10^5^ cells/ml in serum-free media^107^. Transwell inserts (6.5-mm diameter and 8-mm pore size; Corning, Inc.) were loaded with cell suspensions (100 μl on top of each insert), and the lower chambers were filled with either 600 μl of 0.2% FBS supplemented DMEM or 550 μl of 0.2% FBS supplemented DMEM + concentrated trophoblast-CM 50 μl. Cells migrated from upper chamber to lower chambers were stained with crystal violet (Sigma) before being imaged at 24 h and 48 h of 37 °C incubation. Counts were made on seven samples per group for analysis. To count the number of cells passing through the membrane, we used a 20 × magnification. At least five distinct experiments were conducted for each treatment.

### In vitro three-dimensional tube formation assay

In order to investigate the effect of *cis* P-tau aggregation on endovascular activity of first trimester human trophoblasts (HTR-8 cell line) and endothelial cells (HUVEC), we have performed a serum-based three-dimensional dual cell culture experiment as described^52,78^. Human umbilical vein endothelial cells (HUVECs) were cultured in EBM-2 medium (Lonza, Catalog #: CC-3156). All cells were maintained in standard culture conditions of 5% CO_2_ at 37 °C and subjected to three-dimensional tube formation assay as described^9^. *cis* mAb, *trans* mAb, and IgG antibodies were used at 100 ng/ml in the tube formation experiment. We utilized 10% v/v of normal pregnancy serum (NPS) or early preeclampsia serum (e-PES) for majority of investigations, unless otherwise noted. Two independent investigators counted the number of tube-like structures created by the coupled capillary bridge in four separate fields (4 × magnifications) to determine the average number of tubes/vacuoles formed.

### Cellular and tissue confocal microscopy

Confocal microscopy was performed with Nikon A1R (Nikon, Japan) with a 60× or 100× oil immersion objective. For immunofluorescence, PHTs or ADTs (ATG4B^C74A^) were seeded at 400,000 cells/well onto poly-L-lysine coated glass cover slips in 24-well plates. After treatment with 10% PES or 10% NPS or exposure to hypoxia or normoxia, cells were fixed with 4% para-formaldehyde (4% PFA) for 10 min, and then processed in the following order: 2% glycine (10 min), 0.025% Triton X-100 (5 min), PBST (3 times for washing), 10% goat serum B (10 min), and primary antibodies (Supplementary list) in TBST with 4% goat serum (overnight, 4 °C). Coverslips were washed three times with PBST and incubated for 2 h in the dark with secondary antibodies (Alexa Fluor 488 or 594, in PBST with 4% goat serum). Finally, cells were washed with PBS three times before mounting on glass slides with Vecta Shield media (Vector Labs, CA) with or without 4’,6-diamidine-2’-phenylindole dihydrochloride nuclear counterstain. Images were processed with Nikon A1R software followed by ImageJ and Adobe Photoshop®.

Upon delivery, human placental tissue samples underwent two stages of formalin fixation and embedded in paraffin blocks. Placental sections were sequentially de-paraffinized, and treated with 0.1% Sudan Black B (Sigma, Cat# 199,664) for 20 min at room temperature to effectively block autofluorescence. After an extensive wash in phosphate-buffered saline (PBS), the sections were incubated overnight at 4 °C with the primary antibodies (supplementary method list) diluted with Pierce immunostaining enhancer (ThermoFisher Scientific, 46,644) from gestational age-matched normal pregnancy and preeclampsia deliveries. The sections were incubated with the secondary antibodies for 1 h diluted in blocking buffer (2.5% Donkey serum+ 4% BSA + TBST) after a thorough washing in a phosphate-buffered saline (PBS)-Tween 20 solution (PBS: VWR Life Science, 3467C459; Tween-20: Fisher Scientific, BP337-100). Donkey anti-rabbit IgG conjugated to Alexa Fluor 488 (ThermoFisher, A21206) or Alexa Fluor 594 (ThermoFisher, A32754) was used to detect the labeled proteins. At 40X magnification, the mean fluorescence intensity in each section was determined from at least 50 villi in ten randomly chosen fields using ImageJ software (http://imagej.nih.gov/).

### Statistics

All data were analyzed using parametric analysis of variance (ANOVA) or student *t* test. The measurements were taken from different samples. When two groups were compared, the two-tailed *t*-test was used; when many groups were compared, the one-way ANOVA, two way ANOVA or multiple t-tests were used. Prism software was used for statistical analysis (GraphPad Software, Inc). Significant differences were assessed when *p ≤0.05, **p ≤ 0.01, and ***p ≤ 0.001 were used.

### Reporting summary

Further information on research design is available in the Nature Portfolio Reporting Summary linked to this article.

### Supplementary information

Supplementary Information

Reporting Summary

## Data Availability

All data which supports the findings here can be found in the manuscript and supplementary information. Source data for all experiments are provided with this study. Source data are provided with this paper.
