# Supplementary material for: Cis P-tau is a central circulating and placental etiologic driver and therapeutic target of preeclampsia
Source: Nat Commun. 2023 Sep 5;14:5414. doi: 10.1038/s41467-023-41144-6 (PMC10480164; doi:10.1038/s41467-023-41144-6)
Supplement: Supplementary file 1 — Supplementary Information [file 41467_2023_41144_MOESM1_ESM.pdf]

Jash et al., Supplementary Fig.1

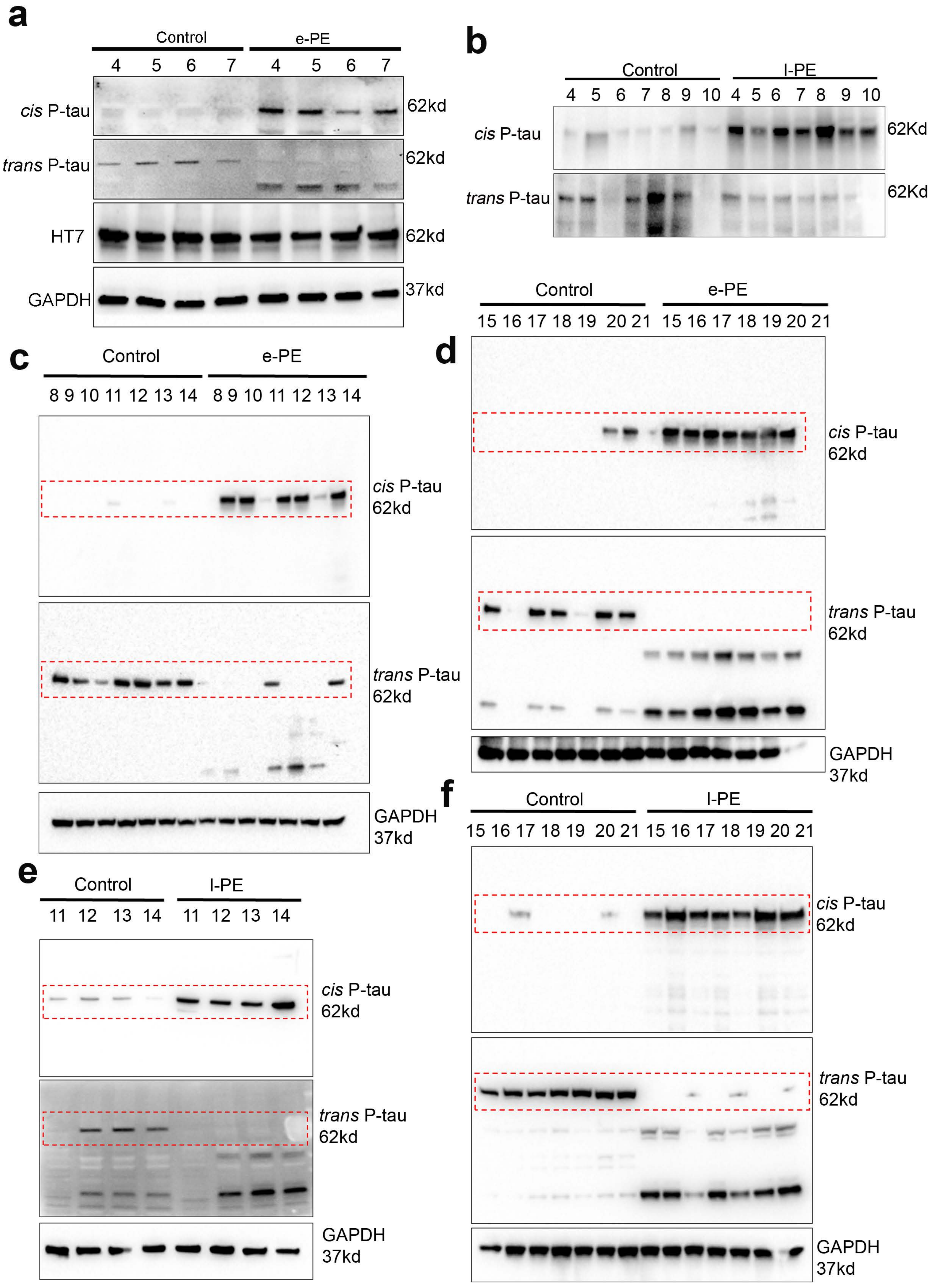

Supplementary Fig. 1. Immunoblot analyses of placental *cis* P-tau, *trans* P-tau and total tau HT7.

a-f, Immunoblotting of *cis* P-tau, *trans* P-tau and HT7 in gestational age-matched control and PE placental protein extracts. Robust *cis* P-tau was observed in the placentas from e-PE (n=21) (**a, c, d**) and l-PE (n=21) (**b, e, f**) vs. respective gestational age-matched controls (n=21 and n=21). Unlike *cis* P-tau, *trans* P-tau was notably absent in both e-PE and l-PE placentas but was expressed in control placental tissues. HT7 remained unaltered across control and e-PE placental tissues (**a**).

Jash et al., Fig. 2

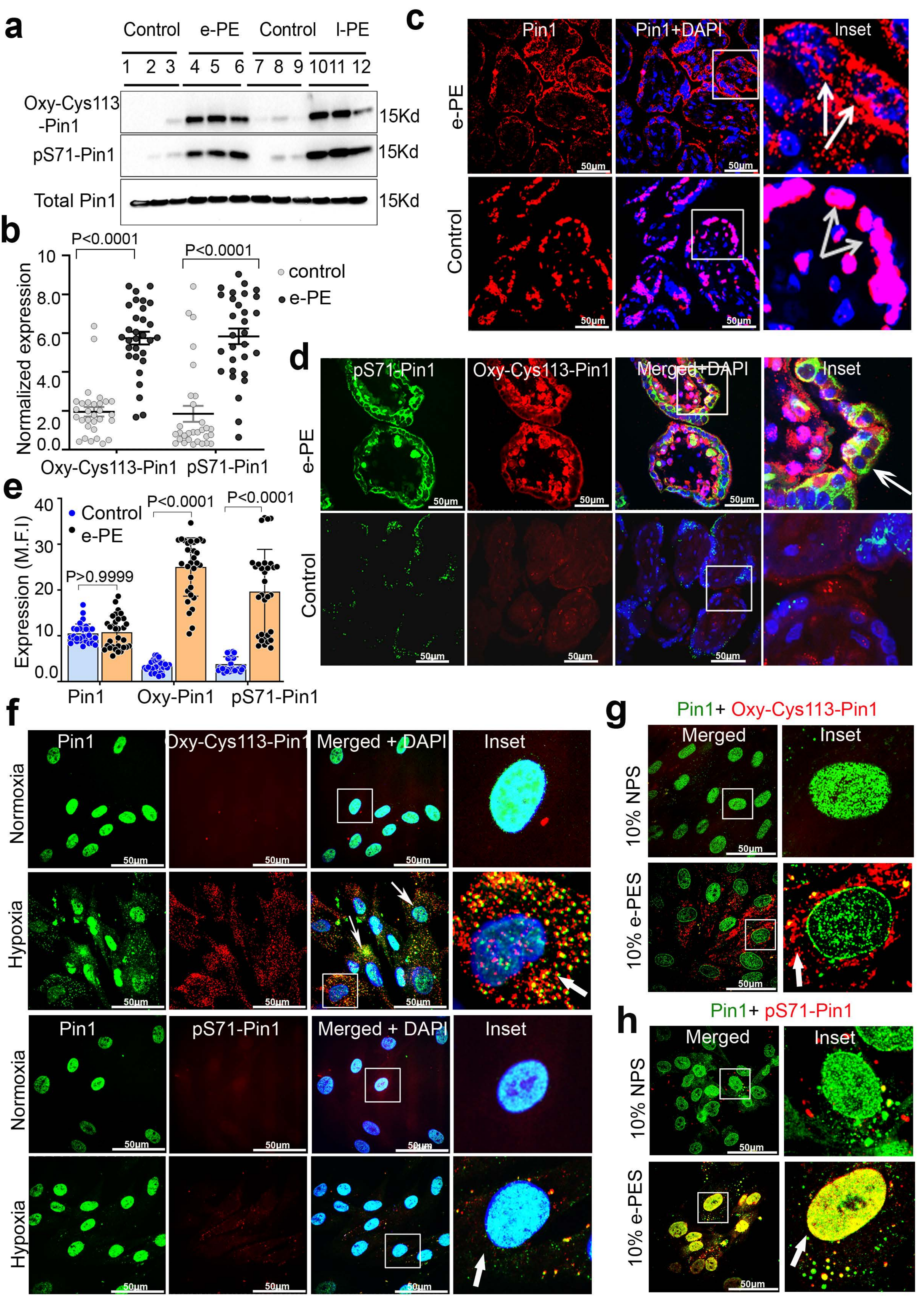

Supplementary Fig. 2. Immunoblot and immunofluorescence analyses of placental *cis* P-tau, *trans* P-tau and total tau HT7.

a, **b,** Expression levels of *cis* P-tau and *trans* P-tau in gestational age-matched control and PE placental protein extracts calculated from immunoblot. Data are presented as mean ± s.e.m and analyzed by two-way ANOVA followed by Bonferroni post-hoc test, n=21 biologically independent samples c**,** Diagram of human placental villous architecture. Symbols: STB, syncytiotrophoblast; CTB, cytotrophoblast; EVT, extravillous trophoblast. **d,** Immunofluorescence detection of tau proteins in gestational age-matched control and PE (e-PE) placental tissues. Tissues were stained for *cis* P-tau, *trans* P-tau, and total tau HT7. Merged images indicated co-localization between *cis* P-tau and HT7 or *trans* P-tau and HT7. Inserts are magnified images of boxed areas in merged images. *Cis* P-tau in e-PE placental tissue and *trans* P-tau in control placental tissue predominantly colocalized with HT7 in the trophoblast layer (arrow). HT7 protein was localized to the entire trophoblast layer as indicated by complete arrow. Scale bar: 100 µm. **e.** Pearson's correlation coefficient for co-localization between *cis* P-tau and HT7 compared to that between *trans* P-tau and HT7 is high but similar (n=12). Data are presented as mean ± s.e.m and analyzed by two tailed unpaired t test with Mann Whitney test.

a. The clinical information of patients with early- and late-onset PE and controls for pathological studies

Variables	Age (years)	BMI (kg/m2)	Gesta-tional age at delivery (weeks)	Maximum systolic blood pressure (mmHg)	Maximum diastolic blood pressure (mmHg)	Maternal hemoglo bin (g/dl)	Maternal platelets ($x10^3/\mu l$)	Aspartate transamin ase (AST) (U/L)	Serum creatinin e (mg/dl)	Urine protein vs creatinine	Birth weight (grams)
Early onset preeclampsia (e-PE; n=17)	32.4 (±6.6)	32.2 (±6.9)	31.6 (±1.4)	184.3 (±17.3)	114 (± 6. 3)	11.3 (±0.79)	101.78 (±56)	128 (±97)	0.96 (±0.71)	7.1 (±6.7)	1298 (±141)
Pre-term birth control (control; n=16)	24.8 (±4.2)	30.08 (±5.7)	31.9 (±1.7)	121.8 (±16.4)	75.4 (±6.9)	11.2 (±1.4)	171.6 (±46)	11 – 30*	0.5 – 1.1*	< 0.3*	1938 (±256)
p-value		†† 0.51[1]	†† 0.11[3]	†† 0.01[3]	†† 0.01[3]	†† 0.34[1]	†† 0.2[1]	--	--	--	††0.02[1]

Variables	Age (years)	BMI (kg/m2)	Gestatio nal age at delivery (weeks)	Maximum systolic blood pressure (mmHg)	Maximum diastolic blood pressure (mmHg)	Maternal hemoglo bin (g/dl)	Maternal platelets (x103/μl)	AST (U/L)	Serum creatinin e (mg/dl)	Urine protein vs creatinine	Birth weight (grams)
Late onset preeclampsia (l-PE; n=19)	29.7 (±9.4)	31.9 (±4.9)	38.7 (±0.69)	174.5(±18 .2)	101.4 (±2.7)	11.6(±2.1)	161(±27. 7)	42.3 (±14.9)	0.87 (±0.18)	3.32 (±3.62)	2891(±50 5.1)
Normal term pregnancy (control; n=21)	29.3 (±7.2)	27.9 (±4.2)	38.4 (±1.4)	117.5 (±9.5)	74.7 (±5.7)	11.3 (±0.8)	225.5 (±62.1)	11 – 30*	0.5 – 1.1*	< 0.3*	3226.7 (±712.9)
p-value		†0.10[1]	† 0.73[3]	† <0.001[3]	† <0.001[3]	† 0.32[1]	† 0.05[1]	--	--	--	† 0.11[1]

Data presented as Mean (standard deviation) for continuous variables.
Data presented as n (%) for categorical variables.
*AST, serum creatinine, and urine protein vs creatinine not measured in Control subjects.
Normal values presented here. Presented as normal ranges.
1t-test (paired). 2 Fisher's exact test. 3 Wilcoxon rank-sum.
† Late onset preeclampsia with severe features versus term controls.
†† Early onset preeclampsia with severe features versus preterm controls.

b. The clinical information of patients with early-onset PE and healthy controls for biomarker studies

Variable	Early onset preeclampsia serum (e-PES; n=38)	Pre-term birth control serum (NPS; n=38)	*p-value*
Age (years)	30.7 (±6.2)	29.1 (±4.6)	NS
BMI (kg/m2)	32.6 (±5.6)	30.7 (±6.2)	†† 0.51[1]
Gestational age at delivery (weeks)	30.4 (±2.7)	29.6 (±3.1)	NS
Maximum systolic blood pressure (mmHg)	179.3 (±19.3)	114.8 (±14.7)	††<0.01[3]
Maximum diastolic blood pressure (mmHg)	117 (± 7. 4)	78.1 (±7.1)	††<0.01[3]
Urine protein vs creatinine	5.3 (±6.2)	< 0.3*	NA
Aspartate transaminase (AST) (U/L)	131 (±89)	11 – 30*	NA
Maternal hemoglobin (g/dl)	11.1 (±1.02)	11.4 (±1.1)	†† 0.34[1]
Maternal platelets ($x10^3/\mu l$)	121.58 (±41)	165.6 (±41)	†† 0.2[1]

Data presented as Mean (standard deviation) for continuous variables.
Data presented as n (%) for categorical variables.
*Serum creatinine, and urine protein:creatinine not mentioned in Control subjects and presented as normal ranges.
NS: not significant, NA: not available
†† Early onset preeclampsia with severe features versus preterm controls.
1t-test (paired). 2 Fisher's exact test. 3 Wilcoxon rank-sum.

Supplementary Fig. 3. The clinical information of PE patients for pathological studies and biomarker studies.

a, The clinical information of patients with early- and late-onset PE and controls for pathological studies. **b,** The clinical information of patients with early-onset PE and healthy controls for biomarker studies. Data presented as Mean ± s.d for continuous variables. (1) paired t-test, (2) Fisher's exact test and (3) non-parametric statistical hypothesis test "Wilcoxon rank sum test".

Jash et al., Supplementary Fig. 4

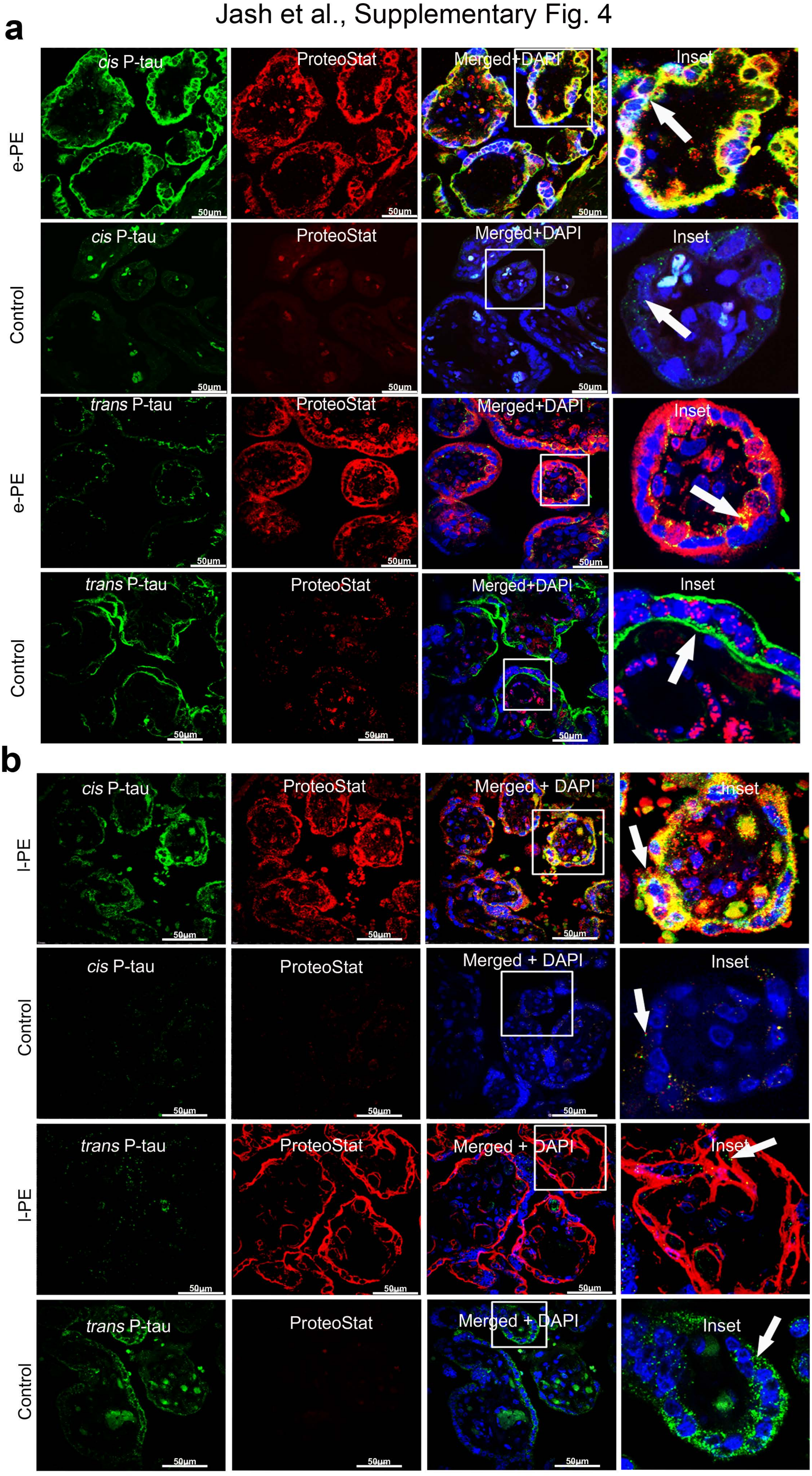

Supplementary Fig. 4. Robust *cis* P-tau expression and aggregation in additional placental tissues from e-PE and l-PE deliveries.

a, Confocal images of human placental tissue sections (n=17; 2-3 different regions of each placenta were analyzed per condition)) from e-PE and its gestational age-matched control (n=16). Placental tissues were subjected to immunofluorescence for *cis* P-tau (green) and ProteoStat (red) or *trans* P-tau (green) and ProteoStat (red). Unlike *trans* P-tau and ProteoStat, the merged picture demonstrated *cis* P-tau co-localization with ProteoStat. Inserts were magnified images of boxed areas in the merged panel. ProteoStat-positive *cis* P-tau aggregates were predominantly detected in the trophoblast layer of the e-PE placenta (indicated by arrow). Unlike *cis* P-tau, the *trans* P-tau signal in the trophoblast layer (arrows) was only seen in control placental tissues (n=16). Scale bar: 50 µm. **b,** Placental tissue sections from l-PE (n=19; 2-3 different regions of each placenta were analyzed per condition) and gestational age-matched control (n=21) deliveries were subjected to double fluorescence for *cis* P-tau and ProteoStat or *trans* P-tau and ProteoStat. Confocal images for double immunofluorescence with *cis* P-tau are shown. The merged image exhibited co-localization of *cis* P-tau and ProteoStat signals. Images of boxed regions were amplified and used as inserts. ProteoStat-positive *cis* P-tau aggregates were mostly seen in the trophoblast layer of the l-PE placenta (arrow). *Trans* P-tau, unlike cis P-tau, was only present in the control placenta's trophoblast layer comprised of both syncytiotrophoblasts and cytotrophobalsts. Scale bar: 50 µm.

Jash et al., Supplementary Fig. 5

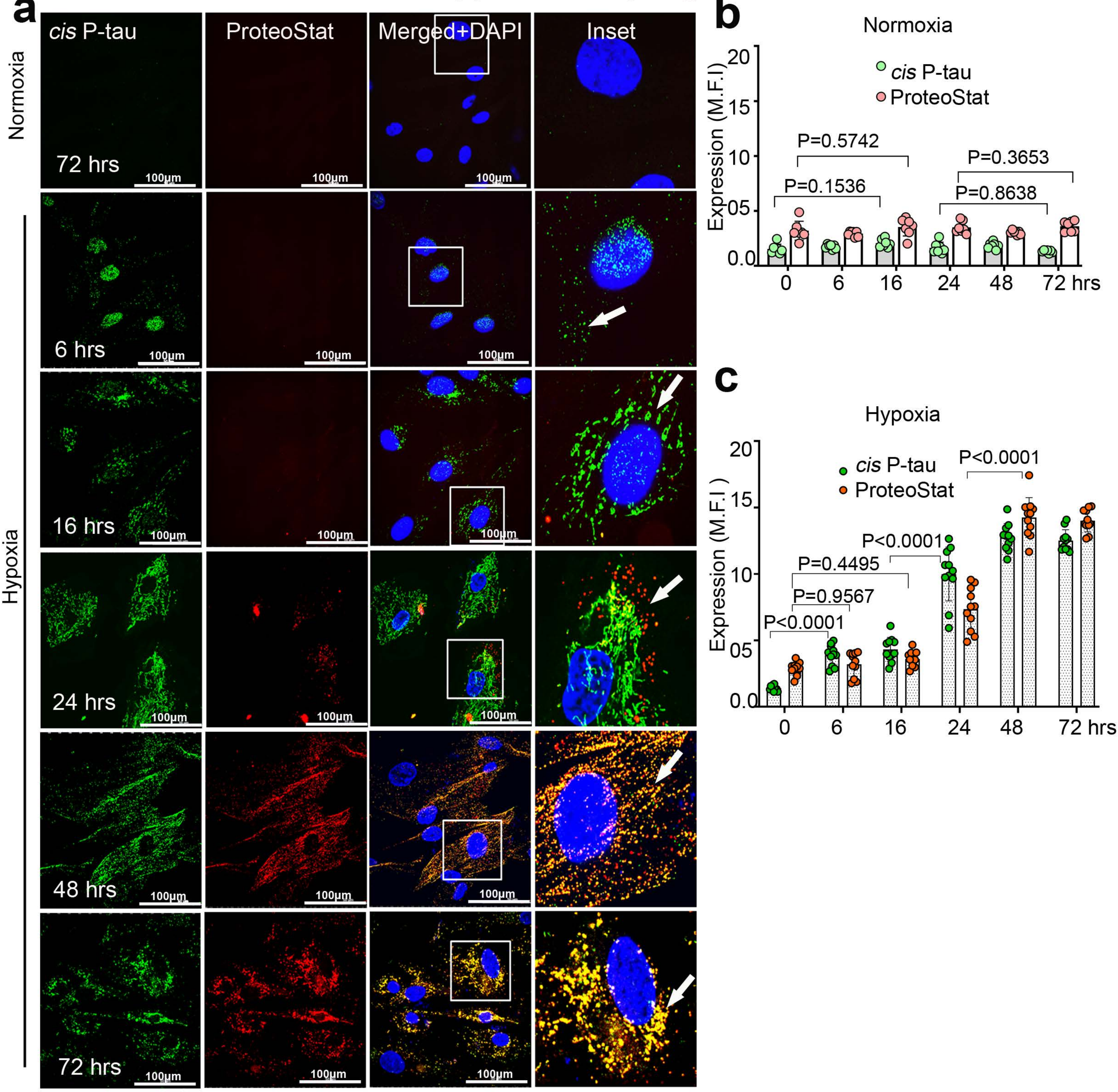

Supplementary Fig. 5. Kinetics of hypoxia-mediated *cis* P-tau induction and its spatiotemporal correlation with protein aggregation.

a, Primary human trophoblasts (PHTs) were grown in serum-free media in a normoxic or hypoxic chamber (1% O_2), fixed at indicated time points and immunostained for *cis* P-tau (green) and ProteoStat dye (red). Confocal immunofluorescence images show single channel (green or red) pictures and merged images along with nuclear DAPI staining. Inset magnified confocal images show co-localization between *cis* P-tau and ProteoStat. Scale bar= 100 µm. **b,** Mean fluorescence intensity (MFI) quantification of *cis* P-tau and ProteoStat during kinetic exposure to normoxia as shown in **a.** A total of n=3 independent experiments with total 2-3 separate fields in the well were analyzed from each group. Statistical significance was evaluated by two-way ANOVA followed by Dunnett's multiple comparisons post hoc test. Data are represented as mean ± s.e.m and show that no *cis* P-tau or ProteoStat was detected. **c,** Mean fluorescence intensity (MFI) quantification of *cis* P-tau and ProteoStat during kinetic exposure to hypoxia as shown in **a**. A total of n=5 independent experiments with 3 separate fields were analyzed from each experiment (total n=11 fields) each group. Two-way ANOVA followed by Sidak's post hoc test were used to quantify the data represented as mean ± s.e.m.

Jash et al., Supplementary Fig. 6

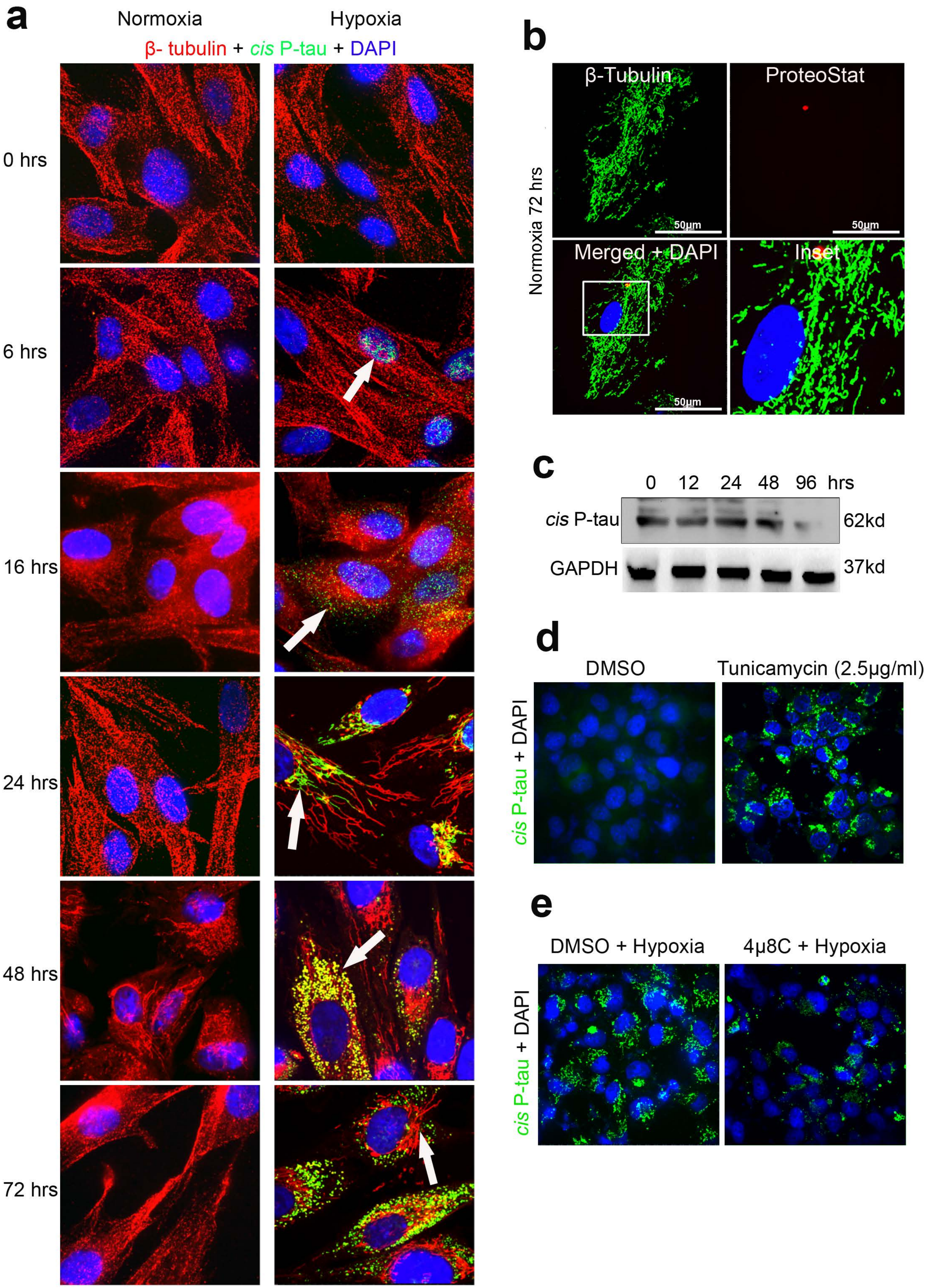

Supplementary Fig. 6. Dynamics of hypoxia-mediated *cis* P-tau induction and β-tubulin disorganization.

a, The dynamic appearance of cis P-tau (green) on the β-tubulin (red) network during hypoxia-induced stress. Cis P-tau first appears in the nucleus during hypoxia (6 hours), followed by cytoplasmic sequestration (16 hrs). At 24 hours, cis P-tau (green) appears on the β-tubulin (red) track and colocalizes. Extended hypoxic stress strongly induces cis P-tau, causing the β-tubulin network to disorganize. Arrowhead points to co-localization between *cis* P-tau and β-tubulin. Scale bar: 100 µm. Even after 72 hours of incubation, normoxia maintains a healthy and organized tubulin-microtubule network in PHTs. Each data point is a representation of n=3 independent experiments. **b,** As described in other figures, normoxia did not induce ProteoStat signal in PHTs. Data represents three separate experiments. Scale bar: 100 µm. **c,** *cis* P-tau persists for more than 48 hr as demonstrated in chase experiments. PHTs were exposed to hypoxia for 72 hr and were transferred to normoxic conditions in chase experiments. The time of transfer to normoxic conditions represented 0 hr followed by chase for varying time points as indicated. Immunoblotting data represents n=3 independent experiments. **d**, PHT's were incubated with ER stress inducer tunicamycin at 2.5 µg/ml or DMSO for 6 hours and immunostained for *cis* P-tau. **e**, PHT's were pretreated with IRE1 inhibitor (4µ8c) (50 µM) for 3 hours prior to hypoxic exposure for 16 hours. and immunostained for *cis* P-tau. Preincubation with 4µ8c significantly reduced hypoxia-induced cis P-tau accumulation. Confocal immunofluorescence images show merged images along with nuclear DAPI staining. Scale bar: 100 µm, each confocal image in d & e is a representation of n=5 independent experiments.

Jash et al., Supplementary Fig. 7

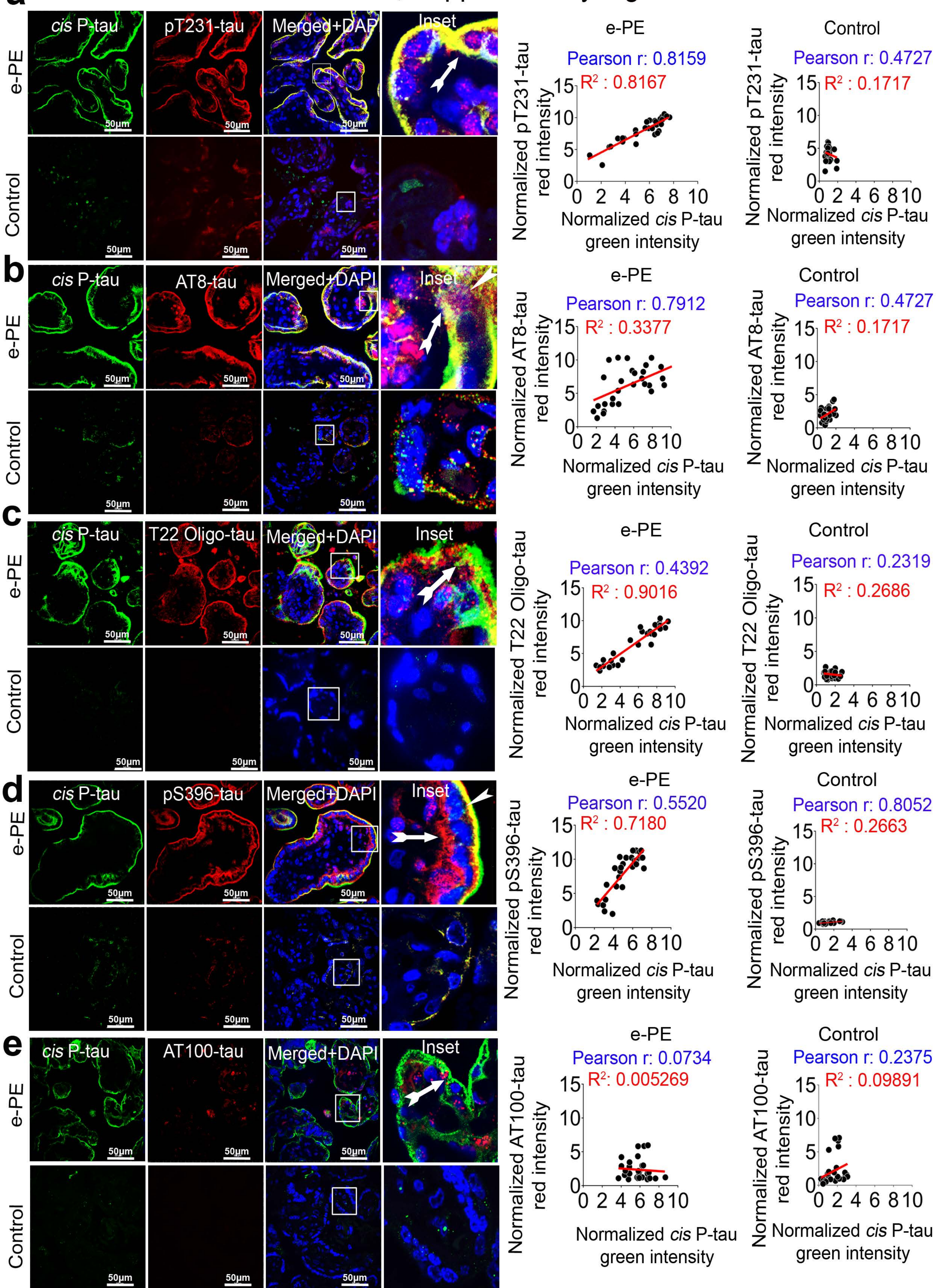

Supplementary Fig. 7. Hierarchical expression of different phosphorylated tau epitopes in e-PE placenta and their colocalization with *cis* P-tau.

e-PE and gestational age-matched (control) placental tissues were analyzed by double immunofluorescence for *cis* P-tau (green) and other hierarchical tau phosphorylation epitopes (red) using respective antibodies. **a**, pT231 (early); **b**, AT8 (intermediate); **c**, oligomeric tau (T22 oligo-tau); **d**, pS396 (late); **e**, AT100 (mature tangle) along with DNA dye DAPI. Merged images indicated co-localization between *cis* P-tau and other tau phosphorylation epitopes. Inserts represent magnified images of boxed areas in each panel. *Cis* P-tau was predominantly detected in the trophoblast layer and co-localized with pT231, AT8, pS396 and T22 oligo-Tau in the e-PE placenta, not in control placenta. Scale bar: 50 µm. On the right side of each image, linear correlation (R^2) between different tau epitopes (red fluorescence intensity) and *cis* P-tau (green fluorescence intensity) across e-PE (n=10) and control (n=11) placental samples is shown. Multiple regions (n=3-4) from each placenta were evaluated to confirm the results. Pearson correlation coefficient (PCC; r) between *cis* P-tau and other tau epitopes was analyzed and shown on top of each graph from Z-stack images of individual placental sections (n=30).

Jash et al., Supplementary Fig. 8

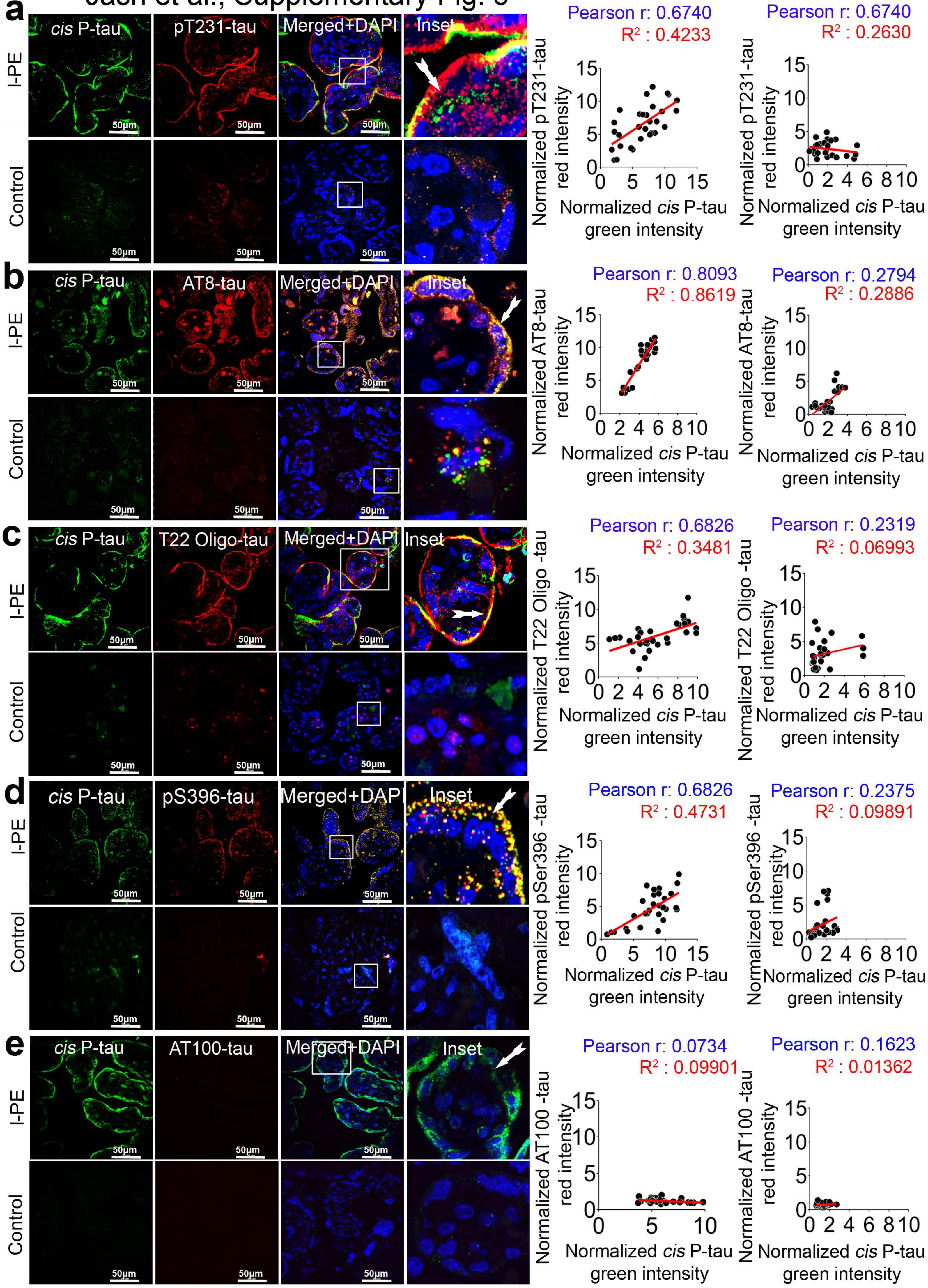

Supplementary Fig. 8. Hierarchical expression of different phosphorylated tau epitopes in l-PE placenta and their colocalization with *cis* P-tau.

As shown in the legend to Supplementary Fig. 7, similar analysis was carried out for *cis* P-tau and other tau phosphorylation epitopes in placental tissue from l-PE and normal pregnancy deliveries. **a**, pT231 (early); **b**, AT8 (intermediate); **c**, oligomeric tau (T22 oligo-Tau); **d**, pS396 (late); **e**, AT100 (mature tangle) along with DNA dye DAPI. Merged images with nuclear DAPI indicated co-localization between *cis* P-tau and other phospho tau epitopes. Different regions (n=3-4) of the placenta from each group l-PE (n=14) and control (n=11) were analyzed and a representative image is shown. The linear correlation (R2) between distinct tau epitopes (red fluorescence intensity) and cis P-tau (green fluorescence intensity) across l-PE and control placental samples is presented on the right side of each image. The Pearson correlation coefficient (r) between cis P-tau and other tau epitopes was calculated and displayed on top of each graph derived from Z-stack images of individual placental sections (n=30).

Jash et al., Supplementary Fig. 9

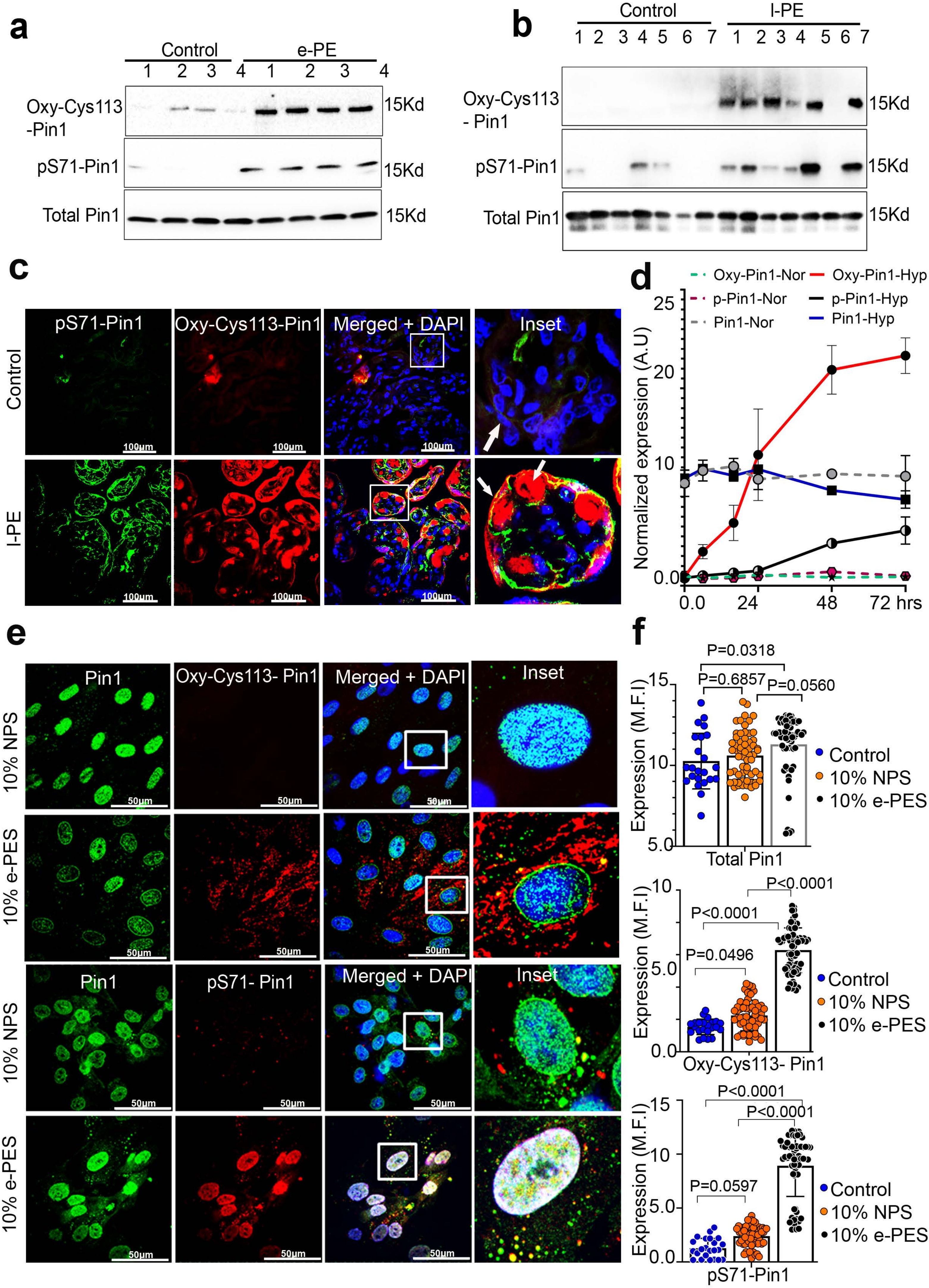

Supplementary Fig. 9. Post translational inactivation of Pin1 triggers pathologic *cis* isomerization in preeclampsia.

a, b, Immunoblotting of pS71 Pin1, Oxy-Cys113 Pin1 and total Pin1 in gestational age -matched control and PE (e-PE & I-PE) placental protein extracts. Expression of inactive forms of Pin1, pS71 Pin1 and Oxy-Cys113 Pin1, was significantly higher in the placentas from e-PE (n=4) and l-PE (n=7) compared to respective gestational age-matched controls (n=4 and n=7). Unlike inactive Pin1 moieties, total Pin1 protein expression remained unaltered in control and PE placentas. **c,** Confocal images of altered Pin1 isoforms in placental tissue from l-PE and control deliveries. Human placental tissue sections from late onset preeclampsia (l-PE) and gestational age-matched controls were immunostained with antibodies for oxidized Pin1 (Oxy-Cys113 Pin1, red) and phospho Pin1 (pS71 Pin1, green) and confocal images were recorded. pS71 Pin1 and Oxy-Cys113 Pin1 are abundant in the l-PE placenta's cytotrophoblast and syncytiotrophoblast layers. Scale bar: 50 µm. **d,** Mean fluorescence intensity (MFI) quantification of oxPin1-Cys113, pPin1-S71 and total Pin1 during kinetic exposure to normoxia or hypoxia as discussed in the Supplementary Fig. 5a-c. Data represent n=3 independent experiments. A total of 2-3 separate fields were analyzed for each group. **e,** Sera from PE patients inactivate physiological Pin1 and induce inactive variants of Pin1 (Oxy-Cys113 Pin1 and pS71-Pin1). PHTs were incubated for 24 hr with serum (10% v/v) from normal pregnancy or early onset preeclampsia pregnancies (e-PE). Preeclampsia serum (e-PES, n=21), not normal pregnancy serum (NPS, n=21) induced inactive Pin1 variants (see insets). Confocal images represent n=21 separate experiments with different serum samples. Scale bar: 100 µm. **f,** Mean fluorescence intensity (MFI) quantification of Pin1, Oxy-Cys113 Pin1 and pS71 Pin1 from experiments described in Fig. 2g, h and Supplementary Fig. 9e**.** Confocal Images are representatives of 21 independent experiments using NPS (n=21), e-PES (n=21), and control (n=23). A total of 61(NPS) and 61 (e-PES) confocal images were analyzed from each group and statistically stratified by one way ANOVA followed by Tukey's post hoc test. The data are represented as mean ± s.e.m.

Jash et al., Supplementary Fig.10

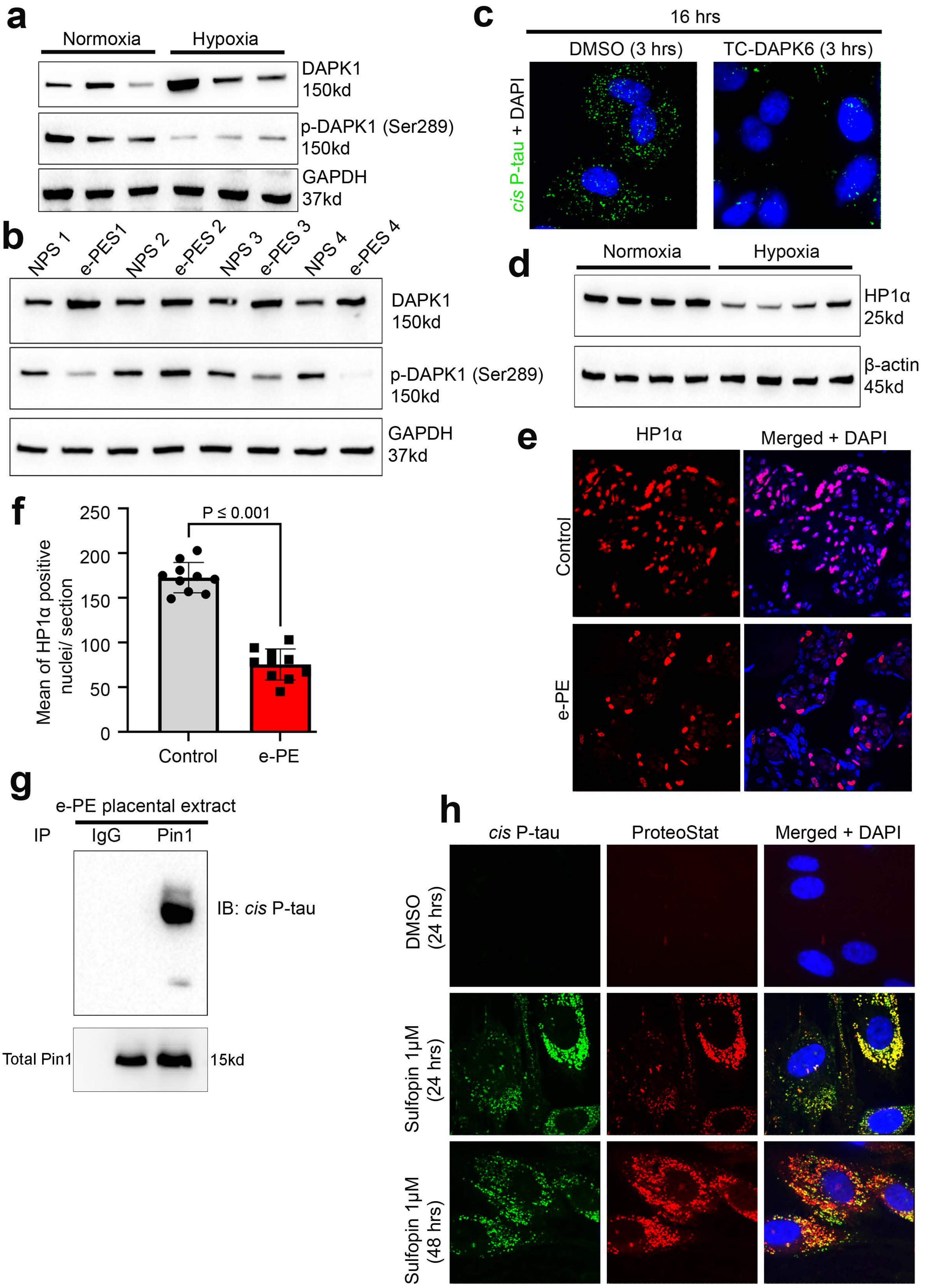

Supplementary Fig. 10. Dynamic regulation of Pin1 in preeclampsia.

a, Immunoblotting reveals that 72 hours of H/R exposure induces total DAPK1 and inhibits pDAPK1 in PHT's. **b**, Total DAPK1 and pDAPK1 immunoblotting was performed on protein extracts from ADTs treated with e-PES (n = 4) and NPS (n= 4) for 24 hours. These experiments represent n=3 biological replicates. **c**, PHT's were pretreated for 3 hours with a DAPK1 inhibitor (TC-DAPK6) before being exposed to hypoxia for 16 hours. and *cis* P-tau immunostained. Preincubation with TC-DAPK6 reduced hypoxia-induced *cis* P-tau accumulation significantly. Confocal immunofluorescence images show merged images along with nuclear DAPI staining. Scale bar: 100 μm. Each image is a depiction of n=3 independent experiments. **d**, Western blot analysis of HP1a after 72 hours of H/R exposure in PHT's, n=3 independent western blot run with n=4 independent biological samples from each group. **e,** Confocal images of nuclear HP1a in placental tissue from e-PE and gestational age-matched controls. **f,** Mean numbers of HP1a positive nuclei from each placental section. n=10 biologically independent placental sections were analyzed for each group. Data are presented as mean ± s.e.m and analyzed by two tailed unpaired t test with Mann Whitney test. **g**, Extract from the placenta (e-PE, n=4) was subjected to IgG or Pin1 pulldown followed by *cis* P-tau immunoblot. **h**, Primary human trophoblasts (PHTs) were treated with 1uM Sulfopin, fixed at indicated time points and immunostained for *cis* P-tau (green) and ProteoStat dye (red). Confocal immunofluorescence images show single channel (green or red) pictures and merged images along with nuclear DAPI staining. Scale bar= 100 μm. Each data point is a representation of n=5 independent experiments.

Jash et al., Supplementary Fig.11

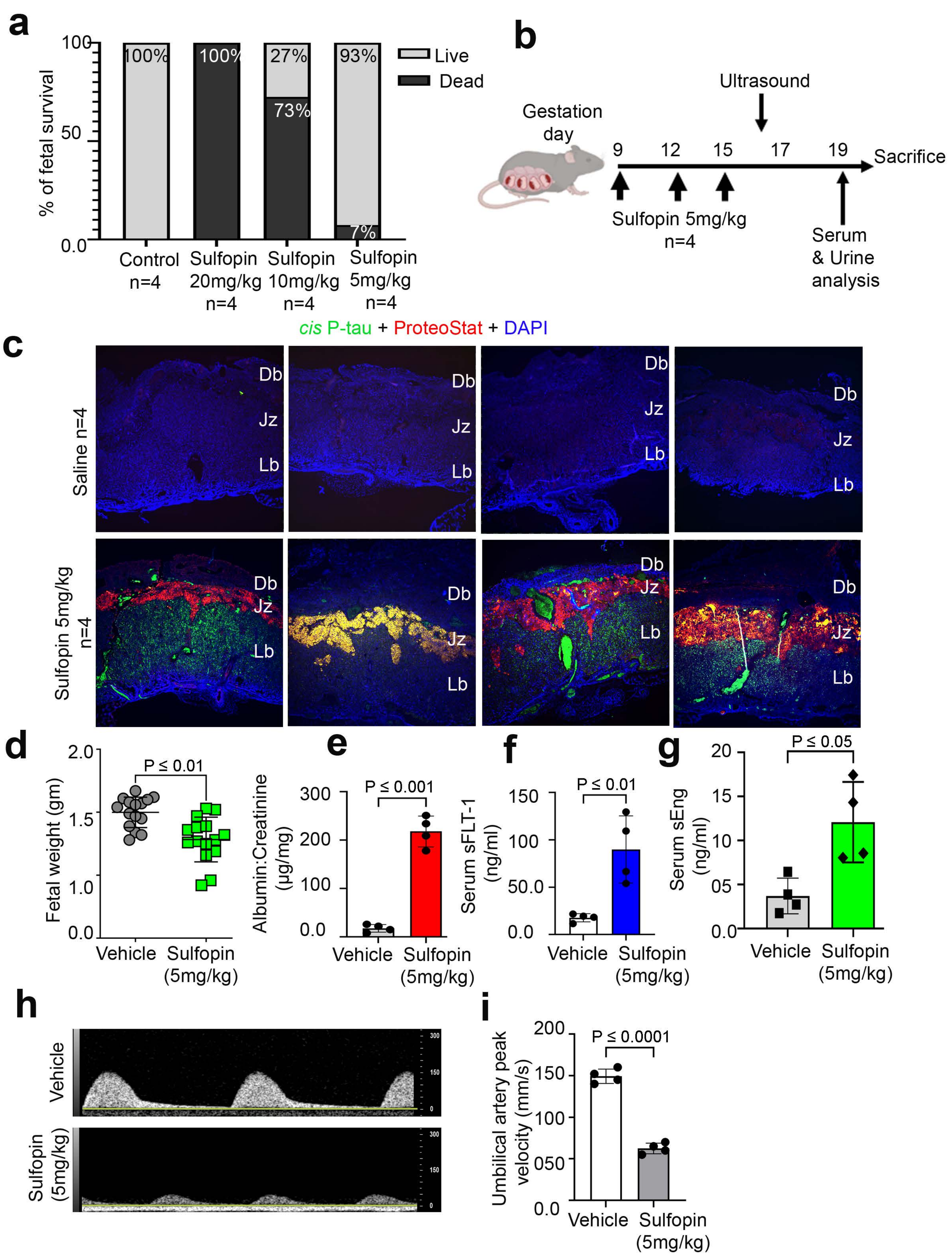

Supplementary Fig. 11. Pin1 inhibition induces PE like features in hTau mice.

Pregnant hTau mice received daily doses of 5 mg/kg (n=4), 10 mg/kg (n=4), 20 mg/kg (n=4) or vehicle (n=4) as a control starting on gestational day (GD) 9 until GD15.**a**, Sulfopin at high doses causes fetal death and results in a poor fetal outcome. **b**, Diagram depicting the hTau pregnant mouse model where mice were treated with Sulfopin (5 mg/kg) or vehicle on gestational days 9, 12, and 15 (n=4).**c**, Representative confocal images of placentas from pregnant hTau mice treated with Sulfopin (5 mg/kg) or vehicle show that Pin1 inhibition induced the highest placental expression of *cis* P-tau in the junctional zone and labyrinth, as well as ProteoStat positive protein aggregation at the junctional zone. **d,** Weight measurements of individual embryos on GD 19 reveal Sulfopin (5 mg/kg) induced intrauterine growth restriction. n=4 mice per group, two-tailed unpaired t-test Mann Whitney test; mean ± s.e.m., p=0.0006. **e**, Spot urine albumin/creatinine ratio evaluations on gd17.5 reveal Sulfopin-induced proteinuria, n=4 mice per group, two-tailed unpaired t test with Welch's correction; mean ± s.e.m., p=0.0007. **f-g**, Measurements of serum sFLT-1 (**f**) and sEng (**g**) concentrations on gd19 reveal Sulfopin-induced elevation of sFLT-1 and sEng. Data are presented as mean ± s.e.m. and analyzed by two-tailed unpaired t test with Mann Whitney test, p= 0.0069 (f), p=0.0286 (g).**h-I**, Representative pulse-wave Doppler ultrasound images (**h**) and analyses of systolic velocities (**i**) of umbilical artery on GD16.5 reveal Sulfopin-induced decreases in umbilical artery systolic velocities , two-tailed unpaired t test with Welch's correction; mean ± s.e.m., p <0.0001.

Jash et al., Supplementary Fig. 12

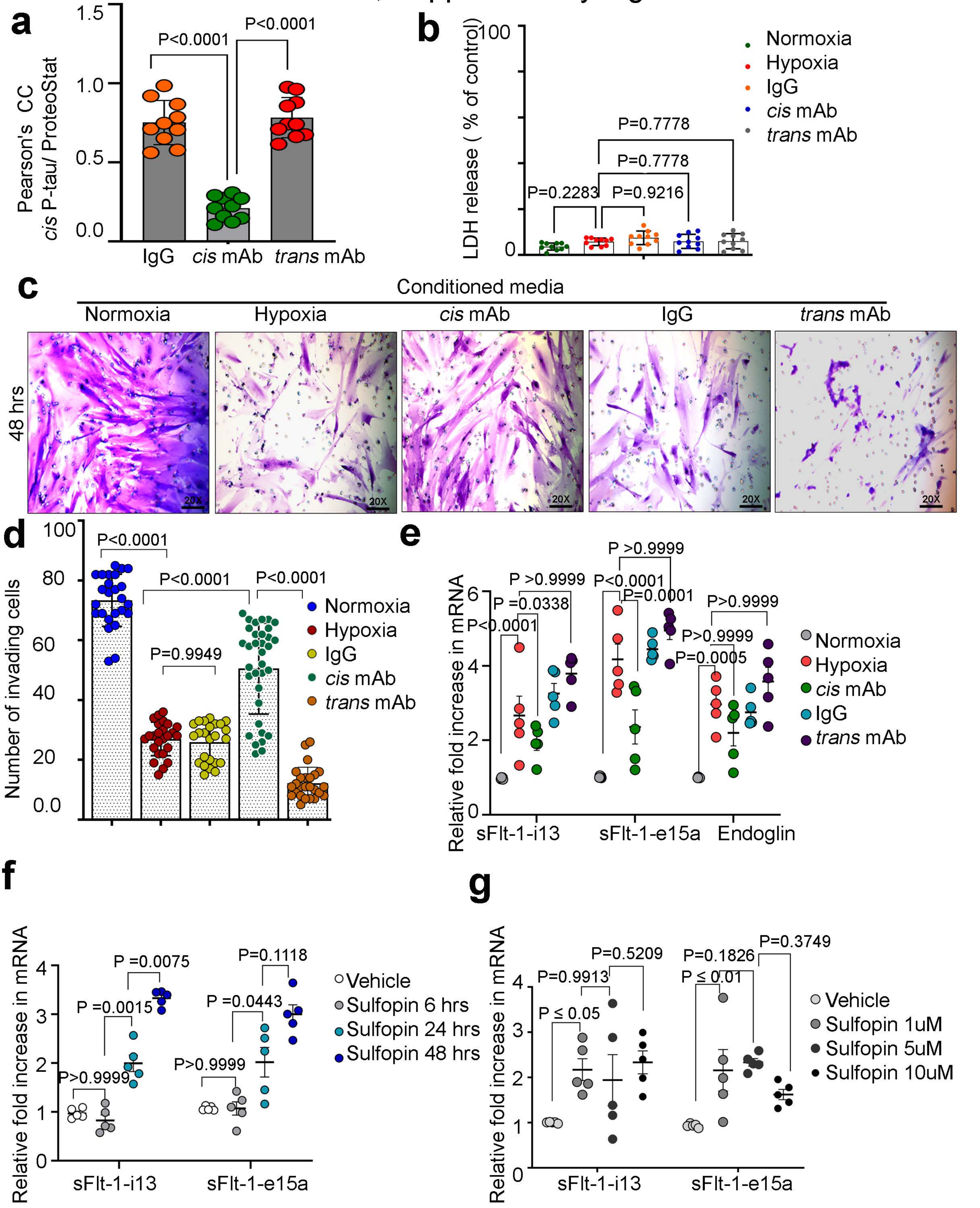

Supplementary Fig. 12. C*is* mAb neutralizes *cis* P-tau, disrupts protein aggregates, and restores human trophoblast invasion and endovascular remodeling.

a, Confocal images from experiment Fig. 3a were analyzed for co-localization between *cis* P-tau and ProteoStat-positive protein aggregates by Pearson's coefficient correlation (n=5). Data are presented as mean ± s.e.m. and analyzed by one way ANOVA followed by Tukey's post hoc tests. **b,** Conditioned media (CM) from Fig. 3a, d were collected and tested for their ability to induce cell death, as determined by the release of lactate dehydrogenase (LDH) into the media. Data represent mean ± s.d. with n=10 biological replicates and analyzed by one way ANOVA followed by Dunnett's multiple comparison post hoc tests. **c,** Three-dimensional (3D) *in vitro* Matrigel invasion assay as shown in Fig. 3e to assess the sustained effect of intracellular *cis* P-tau accumulation and extracellular spread for 48 hr. PHTs were seeded on the upper layer of the Matrigel-coated membrane in the 3D Matrigel chamber and incubated with concentrated CM from experiment Fig. 3a, d for 24 and 48 hours. The representative images were obtained after 48 hr (n=5 independent experiments) and show 20x fields of Matrigel-coated 8 mm pore PET membranes after eliminating all noninvasive cells and staining the membrane with Crystal violet. **d,** the percentage of cells migrating/invading through the Matrigel matrix to reach the underlying membrane is shown. A total of 27-48 separate fields were analyzed from n= 5 independent experiments. One way ANOVA followed by Tukey's post hoc test were applied for assessing statistical significance and the data are presented as mean ± s.e.m. **e,** Effects of hypoxia along with *cis* P-tau mAb, IgG and *trans* P-tau mAb relative to normoxia, on sFlt-1 i13, e15a and endoglin mRNA expression in PHT's. **f, g** After incubation with Sulfopin in PHT's, alternative spliced forms sFlt-1 i13, e15a, and endoglin increased in a time (f) and dose (g) dependent manner. Data (e, f, g) are presented as mean ± s.e.m, from n=5 biologically independent experiments, two-way ANOVA followed by Bonferroni's multiple comparisons test.

Jash et al., Supplementary Fig. 13

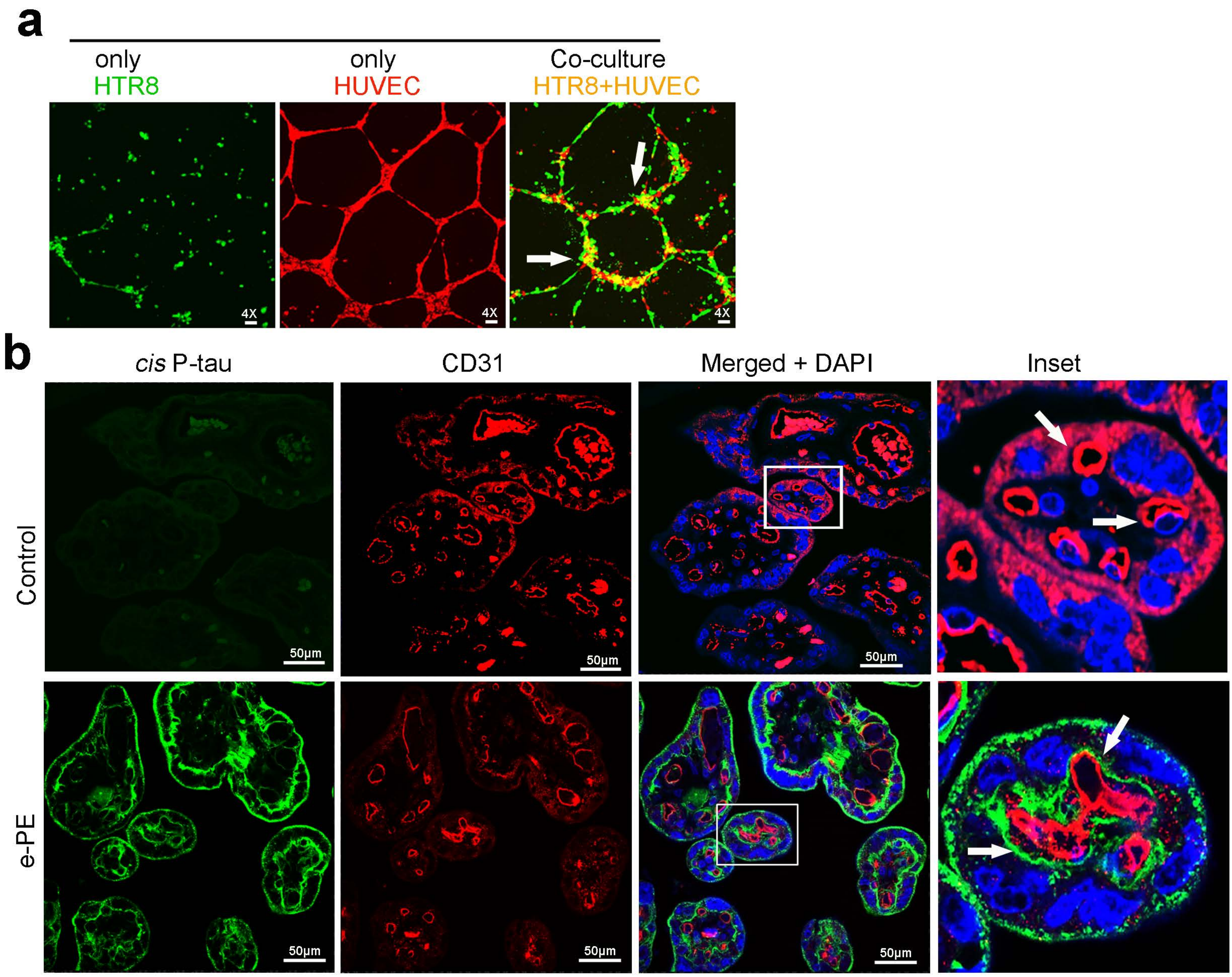

Supplementary Fig. 13. *cis* P-tau positive trophoblast layer and CD31 positive endothelial cells are juxtaposed in the e-PE placenta.

a, 3D vascular tube formation on Matrigel was performed to examine endovascular interaction between CellTracker^{TM} red tagged HUVEC (human umbilical vein endothelial cells) and CellTracker^{TM} green tagged first-trimester trophoblasts (HTR-8 cell line). In contrast to HTR-8 (left), a single cell culture of HUVEC (center) develops 3D tube structures on Matrigel. HTR-8 foot printed on HUVEC for tube formation in a co-culture system (right). **b,** Additional confocal images related to Fig: 3j are shown demonstrating juxtaposition (arrow) of *cis* P-tau positive trophoblast layer and CD31 positive endothelial cells in the e-PE placenta but not in age-matched control placenta (e-PE, n=11; control, n=8).

Jash et al., Supplementary Fig. 14

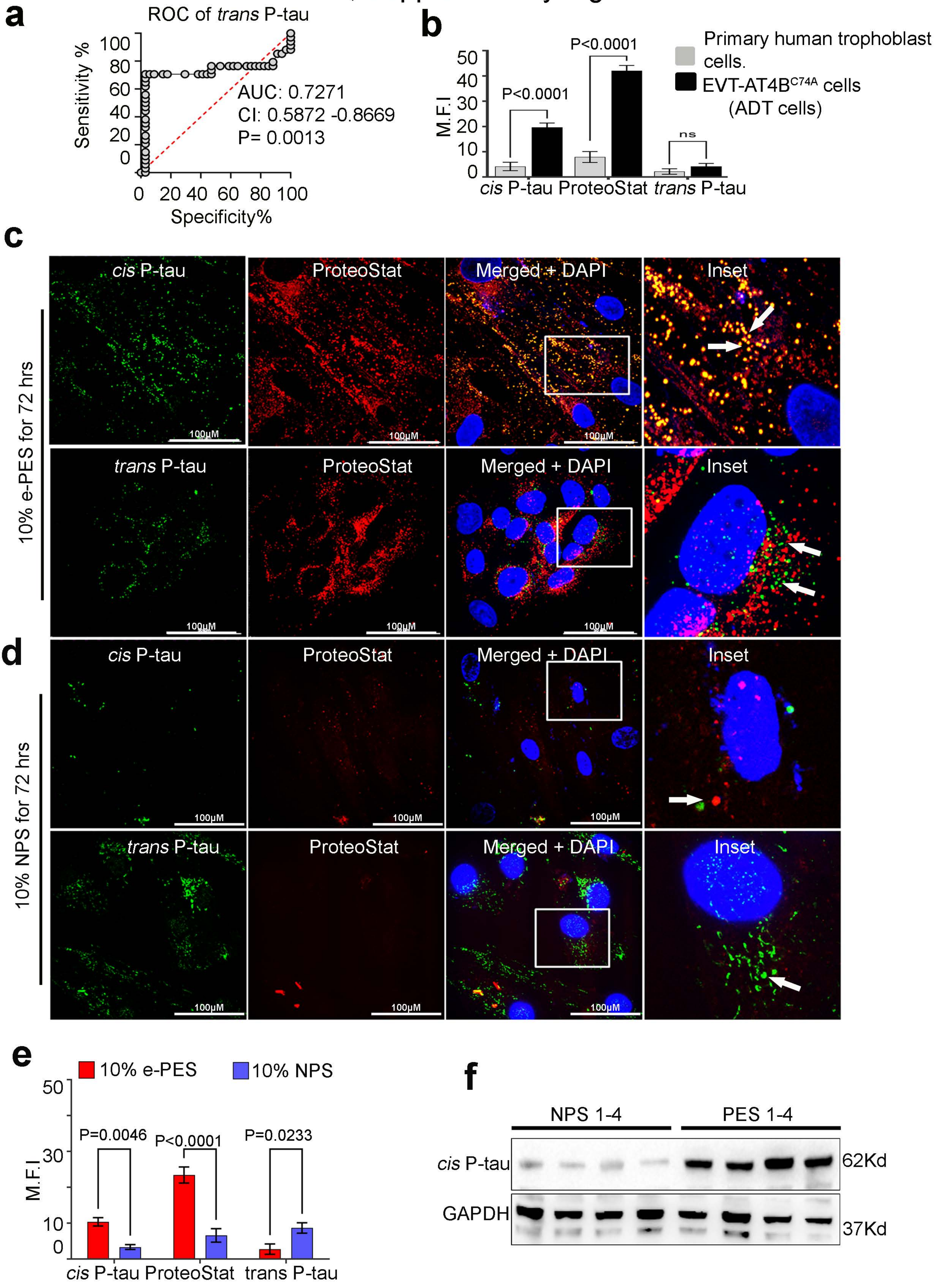

Supplementary Fig. 14. Autophagy deficiency potentiates *cis* P-tau accumulation in human trophoblasts exposed to sera from PE patients.

a, ROC curve analysis of *trans* P-tau abundance in e-PE placenta (n = 38). Area under the ROC curve (AUC) was calculated to be 0.7271 with standard error = 0.03043, 95% confidence interval (CI) 0.5872 to 0.8669 and P value = <0.0013. **b,** Mean fluorescence intensity (MFI) of *cis* P-tau, *trans* P-tau, and ProteoStat-positive protein aggregates in autophagy-deficient trophoblasts (ADTs) and PHTs following 24 hours incubation with PES (n=7) and NPS (n=5). Data are presented as mean ± s.e.m calculated by two-way ANOVA followed by Bonferroni's post hoc test. **c, d,** PHTs were incubated for an extended time (72 hours) with 10% sera from e-PE patients (n = 5) or control pregnant women (n = 5), followed by immune colocalization with *cis* P-tau (green) and ProteoStat (red) or *trans* P-tau (green) and ProteoStat (red). Scale bar: 50 µm. Merged and respective inset image show colocalization. White arrows in the merged images indicate either co-localization of *cis* P-tau and ProteoStat or lack of colocalization of *trans* P-tau and ProteoStat. **e,** Quantification of MFI of *cis* P-tau, *trans* P-tau, and ProteoStat-positive protein aggregates (PES, n=5; NPS, n=5). Data are presented as mean ± s.e.m calculated by two-way ANOVA followed by Šídák's multiple comparisons test. **f,** *Cis* P-tau immunoblotting was performed on protein extracts from ADTs treated with PES (n = 3) and NPS (n= 3) for 24 hours, followed by a chase for 48 hours in media devoid of PES and NPS. These experiments represent n=4 biological replicates.

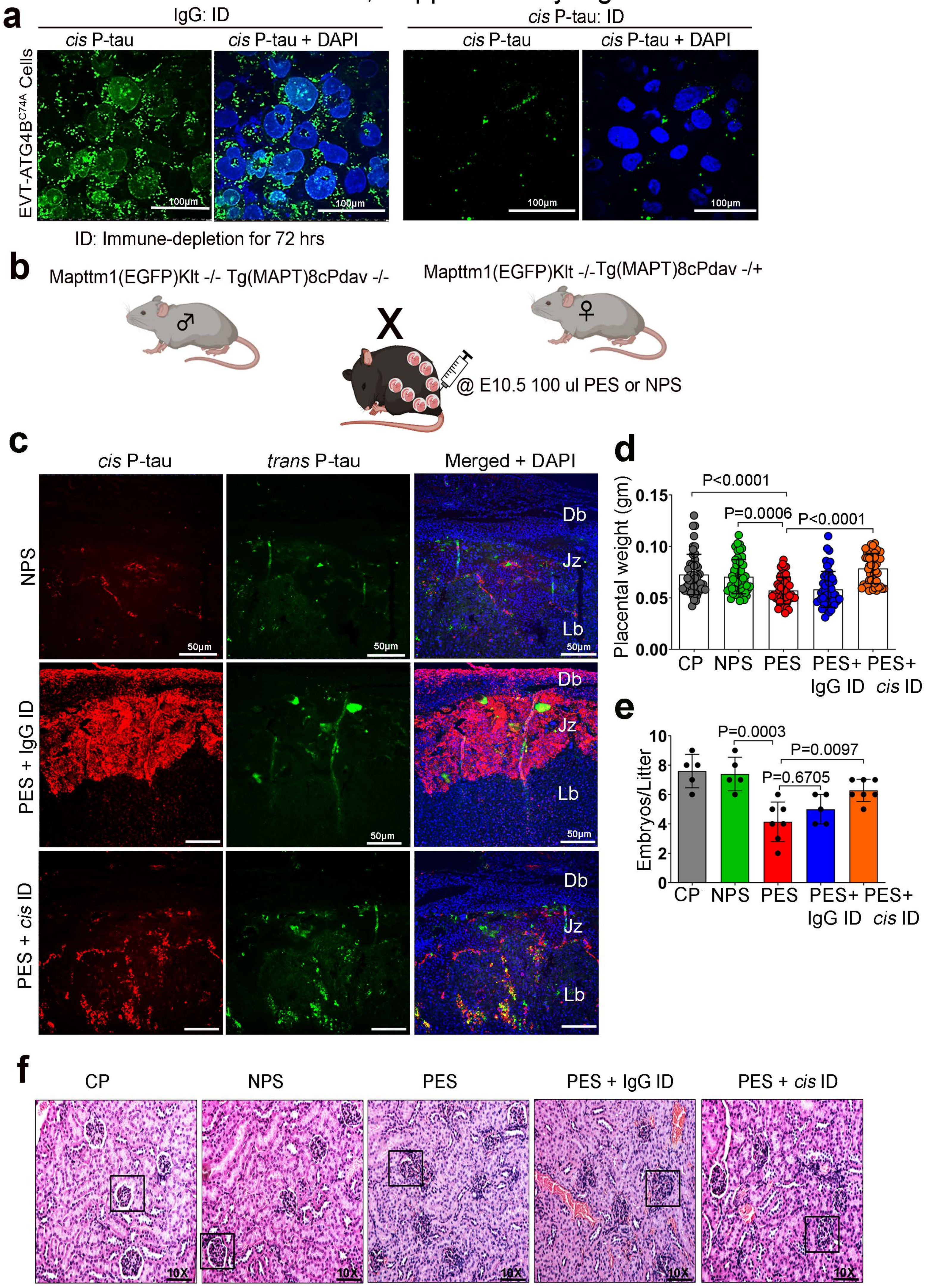
a
IgG: ID
cis P-tau: ID
cis P-tau
cis P-tau + DAPI
EVT-ATG4B^C74A Cells
100µm
ID: Immune-depletion for 72 hrs
b
Mapttm1(EGFP)Klt -/- Tg(MAPT)8cPdav -/-
Mapttm1(EGFP)Klt -/-Tg(MAPT)8cPdav -/+
X
@ E10.5 100 ul PES or NPS
c
cis P-tau
trans P-tau
Merged + DAPI
NPS
PES + IgG ID
PES + cis ID
Db
Jz
Lb
50µm
d
Placental weight (gm)
0.15
0.10
0.05
0.00
P<0.0001
P=0.0006
P<0.0001
CP
NPS
PES
PES+ IgG ID
PES+ cis ID
e
Embryos/Litter
10
8
6
4
2
0
P=0.0003
P=0.0097
P=0.6705
f
CP
NPS
PES
PES + IgG ID
PES + cis ID
10X

Supplementary Fig. 15. *cis* P-tau immune depletion in PE serum effectively blocks PE like pathology in htau mice model of preeclampsia.

a, PES was immunodepleted for 72 hours with *cis* mAb or IgG isotype before being incubated with ADT cells for 24 hr grown in serum-free media. After incubation, cells were fixed and immunostained for *cis* P-tau. The nuclei were stained with DAPI (blue). Scale bar: 20 µm. Representative confocal images (n=10) showed efficient and significant *cis* P-tau depletion from PES by *cis* mAb. n=5 biologically independent PES was used for immunodepletion, each experiment was repeated n=3 times. **b,** htau model of preeclampsia. Female htau mice with homozygous for the targeted mutation (Mapttm) and hemizygous for the transgene (TgMAPT) were mated with male htau mice with homozygous for the targeted mutation and noncarrier for the transgene. The day of vaginal plug appearance was designated gestational day (gd) 0. On gd 10, 100 ul PES or NPS were injected i.p. to induce PE-like features. **c,** Extended confocal images of Fig: 5c. Placentas from NPS, PES + IgG ID and PES + *cis* ID-treated htau dams were double immunostained for *cis* P-tau and *trans* P-tau. The placental expression of *cis* P-tau was the strongest in the junctional zone and decidia basalis in PES -treated group. *cis* P-tau depletion significantly reduced *cis* P-tau induction in the junctional zone, multiple placental sections (n=5-7). **d,** Individual placental weight on GD17.5 was presented in response to various treatments, as stated. PES resulted in considerable placental weight loss and *cis* P-tau depletion effectively reversed this loss. CP (n=5) NPS (n=5), PES (n=10), PES + IgG ID (n=10), PES+ cis ID (n=7). The statistical significance of the data was determined by one way ANOVA followed by Tukey's post hoc test; mean s.e.m. **e,** Total number of embryos per litter on gd 17.5 in response to various treatments, as mentioned for CP (n=5) NPS (n=5), PES (n=10), PES + IgG ID (n=5), PES+ cis ID (n=7). Statistical significance of the data was determined one way ANOVA followed by Tukey's post hoc test; mean s.e.m. **f,** Representative images of H&E-stained histo-pathological analysis of sagittal kidney sections from the indicated groups showing normal glomeruli (black arrow) and glomerular endotheliosis (yellow arrow).

Supplementary Fig. 16. A list of the antibodies used and their specific applications.

Antibody	Vendor	Catalogue#	Dilution
cis P-tau mAb	Lab generated[37,39]		1:200 (Immunofluorescence)<br>1:1000 (Western blotting)
trans P-tau mAb	Lab generated[37,39]		1:200 (Immunofluorescence)<br>1:1000 (Western blotting)
Oxy-Cys113-Pin1 mAb	Lab generated[38]		1:500 (Immunofluorescence)<br>1:1000 (Western blotting)
pS71-Pin1 mAb	Lab generated[68]		1:500 (Immunofluorescence)<br>1:1000 (Western blotting)
Pin1- Rabbit mAb	Cell signaling	3722	1:1000 (Immunofluorescence)<br>1:2000 (Western blotting)
phospho T231 mAb	Abcam	ab151559	1:300 (Immunofluorescence)
Phospho-Tau (Ser202, Thr205)-AT8 mAb	ThermoFisher	MN1020	1:100 (Immunofluorescence)
Anti-phospho-Tau (pSer396), Rabbit polyclonal	Millipore Sigma	SAB4504557	1:50 (Immunofluorescence)
Anti-Tau(T22), oligomeric tau, Rabbit polyclonal	Millipore Sigma	ABN454	1:50 (Immunofluorescence)
Phospho-Tau (Thr212,Ser214) (AT100) mAb	ThermoFisher	MN1060	1:250 (Immunofluorescence)
Anti-CD31 mAb	Abcam	ab182981	1:500 (Immunofluorescence)
Anti-HP1α mAb	Cell signaling	2616	1:1000 (Western blotting)<br>1:100 (Immunofluorescence)
Anti-DAPK1 Antibody	Cell signaling	3008	1:1000 (Western blotting)
Anti-Phospho-DAPK1 (Ser289)	ThermoFisher	PA5-105873	1:1000 (Western blotting)
Donkey anti-Rabbit IgG (H+L) Highly Cross-Adsorbed Secondary Antibody, Alexa Fluor™ 488	ThermoFisher	A-21206	1:1000 (Immunofluorescence)
Donkey anti-Rabbit IgG (H+L) Highly Cross-Adsorbed Secondary Antibody, Alexa	ThermoFisher	A-21207	1:1000 (Immunofluorescence)

Fluor™ 594			
Donkey anti-Mouse IgG (H+L) Highly Cross-Adsorbed Secondary Antibody, Alexa Fluor™ 594	ThermoFisher	A-21203	1:1000 (Immunofluorescence)
Donkey anti-Mouse IgG (H+L) Highly Cross-Adsorbed Secondary Antibody, Alexa Fluor™ 488	ThermoFisher	A-21202	1:1000 (Immunofluorescence)
Goat anti-Mouse IgG (H+L) Secondary Antibody, HRP	ThermoFisher	32430	1:10000 (Western blotting)
Goat anti-Rabbit IgG (H+L) Secondary Antibody, HRP	ThermoFisher	32460	1:10000 (Western blotting)
Rabbit IgG Isotype Control	ThermoFisher	31235	Blocking, Co-IP
Mouse IgG Isotype Control	ThermoFisher	31903	Blocking, Co-IP
