# Supplementary material for: Cis P-tau is a central circulating and placental etiologic driver and therapeutic target of preeclampsia
Source: Nat Commun. 2023 Sep 5;14:5414. doi: 10.1038/s41467-023-41144-6 (PMC10480164; doi:10.1038/s41467-023-41144-6)
Supplement: Supplementary file 2 — Reporting Summary [file 41467_2023_41144_MOESM2_ESM.pdf]

nature portfolio

Corresponding author(s): Sharma, Lu and Zhou

Last updated by author(s): July 21, 2023

Reporting Summary

Nature Portfolio wishes to improve the reproducibility of the work that we publish. This form provides structure for consistency and transparency in reporting. For further information on Nature Portfolio policies, see our Editorial Policies and the Editorial Policy Checklist.

Please do not complete any field with "not applicable" or n/a. Refer to the help text for what text to use if an item is not relevant to your study.
For final submission: please carefully check your responses for accuracy; you will not be able to make changes later.

Statistics

For all statistical analyses, confirm that the following items are present in the figure legend, table legend, main text, or Methods section.

n/a	Confirmed	
[ ]	[X]	The exact sample size (n) for each experimental group/condition, given as a discrete number and unit of measurement
[ ]	[X]	A statement on whether measurements were taken from distinct samples or whether the same sample was measured repeatedly
[ ]	[X]	The statistical test(s) used AND whether they are one- or two-sided<br>*Only common tests should be described solely by name; describe more complex techniques in the Methods section.*
[X]	[ ]	A description of all covariates tested
[X]	[ ]	A description of any assumptions or corrections, such as tests of normality and adjustment for multiple comparisons
[ ]	[X]	A full description of the statistical parameters including central tendency (e.g. means) or other basic estimates (e.g. regression coefficient) AND variation (e.g. standard deviation) or associated estimates of uncertainty (e.g. confidence intervals)
[ ]	[X]	For null hypothesis testing, the test statistic (e.g. F, t, r) with confidence intervals, effect sizes, degrees of freedom and P value noted<br>*Give P values as exact values whenever suitable.*
[X]	[ ]	For Bayesian analysis, information on the choice of priors and Markov chain Monte Carlo settings
[X]	[ ]	For hierarchical and complex designs, identification of the appropriate level for tests and full reporting of outcomes
[X]	[ ]	Estimates of effect sizes (e.g. Cohen's d, Pearson's r), indicating how they were calculated

Our web collection on statistics for biologists contains articles on many of the points above.

Software and code

Policy information about availability of computer code

Data collection	Nikon AIR Confocal High-Content Imaging System, Nikon Fluorescence Microscope, Vevo 3100 High Resolution Ultrasound VisualSonic, NanoDrop 100 Sectrometer, Applied Biosystems 7500 Real Time PCR software.
Data analysis	ImageJ software, Nikon A1R software, GraphPad Prism 9.1, RStudio 4.0.2. Image J was used to quantify the relative intensity of IBs. Nikon A1R software was used to integrate multiple confocal images for visualization and Z-stack imaging. GraphPad Prism 9.1 was used for data annotation and statistical analyses

For manuscripts utilizing custom algorithms or software that are central to the research but not yet described in published literature, software must be made available to editors and reviewers. We strongly encourage code deposition in a community repository (e.g. GitHub). See the Nature Portfolio guidelines for submitting code & software for further information.

Data

Policy information about availability of data

All manuscripts must include a data availability statement. This statement should provide the following information, where applicable:

- Accession codes, unique identifiers, or web links for publicly available datasets
- A description of any restrictions on data availability
- For clinical datasets or third party data, please ensure that the statement adheres to our policy

Research involving human participants, their data, or biological material

Policy information about studies with human participants or human data. See also policy information about sex, gender (identity/presentation), and sexual orientation and race, ethnicity and racism.

Reporting on sex and gender	This study includes term and pre-term pregnancy, as well as early and late onset preeclampsia. Throughout the investigation, only female sex was taken into account.
Reporting on race, ethnicity, or other socially relevant groupings	Following racial combinations was observed in the patient's population related to Extended Fig. 3a, b.<br>Early onset preeclampsia: White (40%), Black (20%), Hispanic (40%), Others (0%).<br>Pre-term birth control: White (20%), Black (40%), Hispanic (40%), Others (0%).<br>Late onset preeclampsia: White (50%), Black (30%), Hispanic (20%), Others (0%).<br>Normal term pregnancy: White (70%), Black (20%), Hispanic (10%), Others (0%).
Population characteristics	The population and clinical characteristics of the patients analyzed are presented in Extended Fig. 3a, b
Recruitment	Participants from Europe and USA were recruited for these studies.
Ethics oversight	All protocols concerning the use of human material were approved by the Institutional review board of Women and Infants Hospital. All subjects gave their informed consent prior to participating in the study.

Note that full information on the approval of the study protocol must also be provided in the manuscript.

Field-specific reporting

Please select the one below that is the best fit for your research. If you are not sure, read the appropriate sections before making your selection.

[X] Life sciences   [ ] Behavioural & social sciences   [ ] Ecological, evolutionary & environmental sciences

For a reference copy of the document with all sections, see nature.com/documents/nr-reporting-summary-flat.pdf

Life sciences study design

All studies must disclose on these points even when the disclosure is negative.

Sample size	According to power calculations based on the two-sample t-test with a two-sided 0.05 significance level, we calculated that a sample size of 8 in each group will provide 80% power to detect a 1.5 standard deviation difference in means of the outcomes to be studied (effect size = 1.5). 10-12 mice per treatment for hTau mouse preeclampsia mouse model; 4-5 mice per arm for Sulfopin;. The size of animal studies was also included in the figure legends. Sample sizes were chosen as large as possible while taking into account the experimental effort required to generate the respective data.
Data exclusions	No data was excluded from the experiments.
Replication	All experimental findings were reliably reproduced at least 5-7 times. All the in vitro experiments were repeated in multiple independent experiments and showed similar results.
Randomization	Human patients were allocated depending on their clinical features and gestational age. For all in vivo experiments, mice were randomly assigned into different treatment arms.
Blinding	Immunofluorescence staining, ultrasound imaging and Confocal image analysis were performed in a blinded fashion. During western blot and pull-down evaluations, investigators were not blinded for two distinct groups in order to load each group together (for example, groups 1 and 2). However, the investigators were unaware of the identity of the contents in each group.

Behavioural & social sciences study design

All studies must disclose on these points even when the disclosure is negative.

Study description	N/A
Research sample	N/A
Sampling strategy	N/A
Data collection	N/A
Timing	N/A
Data exclusions	N/A
Non-participation	N/A
Randomization	N/A

Ecological, evolutionary & environmental sciences study design

All studies must disclose on these points even when the disclosure is negative.

Study description	N/A
Research sample	N/A
Sampling strategy	N/A
Data collection	N/A
Timing and spatial scale	N/A
Data exclusions	N/A
Reproducibility	N/A
Randomization	N/A
Blinding	N/A

Did the study involve field work? ☐ Yes ☒ No

Field work, collection and transport

Field conditions	
Location	
Access & import/export	
Disturbance	

Reporting for specific materials, systems and methods

We require information from authors about some types of materials, experimental systems and methods used in many studies. Here, indicate whether each material, system or method listed is relevant to your study. If you are not sure if a list item applies to your research, read the appropriate section before selecting a response.

Materials & experimental systems

n/a	Involved in the study	
☐	☒	Antibodies
☐	☒	Eukaryotic cell lines
☒	☐	Palaeontology and archaeology
☐	☒	Animals and other organisms
☐	☒	Clinical data
☒	☐	Dual use research of concern
☒	☐	Plants

Methods

n/a	Involved in the study	
☒	☐	ChIP-seq
☒	☐	Flow cytometry
☒	☐	MRI-based neuroimaging

Antibodies

Antibodies used

cis P-tau mAb (Lab generated), trans P-tau mAb (Lab generated), Oxy-Cys113-Pinl mAb (Lab generated) pS71-Pinl mAb (Lab generated), Pinl- Rabbit polyclonal (Cell signaling 3722), phospho T231 mAb- EPR2488 (Abeam ab151559), Phospho-Tau (Ser202, Thr205)-AT8 mAb (ThermoFisher MN1020), Anti-phospho-Tau (pSer396), Rabbit polyclonal (Millipore Sigma SAB4504557), Anti-Tau(T22), oligomeric tau, Rabbit polyclonal (Millipore Sigma ABN454), Phospho-Tau (Thr212,Ser214) (ATl00) mAb ThermoFisher MN1060), Anti-CD31 mAb-EPR17259 (Abeam ab182981), Anti-HPl Rabbit polyclonal (Cell signaling 2616), Anti-DAPKl Antibody- Rabbit polyclonal (Cell signaling 3008), Anti Phospho-DAPKl (Ser289)- Rabbit polyclonal (ThermoFisher PA5-105873), Goat anti-Rabbit IgG (H+L), HRP (ThermoFisher- 32460),Rabbit anti-Mouse IgG (H+L), and HRP (1:2000; ThermoFisher Catalog # PA1-28568). Antibodies details were provided in Methods.

Validation

Except cis P-tau mAb (Lab generated), trans P-tau mAb (Lab generated), Oxy-Cys113-Pinl mAb (Lab generated) and pS71-Pinl mAb (Lab generated), all the other antibodies are commercially available and have been tested to recognize for both human and mouse species. The quality control and validation are provided by the manufacturer's websites:

Pinl- Rabbit polyclonal (Cell signaling 3722): https://www.cellsignal.com/products/primary-antibodies/pin1-antibody/3722
phospho T231 mAb- EPR2488 (Abeam ab151559): https://www.abcam.com/products/primary-antibodies/tau-phospho-t231-antibody-epr2488-ab151559.html
Phospho-Tau (Ser202, Thr205)-AT8 mAb (ThermoFisher MN1020): https://www.thermofisher.com/antibody/product/Phospho-Tau-Ser202-Thr205-Antibody-clone-AT8-Monoclonal/MN1020
Anti-phospho-Tau (pSer396), Rabbit polyclonal (Millipore Sigma SAB4504557): https://www.sigmaaldrich.com/US/en/product/sigma/sab4504557
Anti-Tau(T22), oligomeric tau, Rabbit polyclonal (Millipore Sigma ABN454): https://www.sigmaaldrich.com/US/en/product/mm/abn454
Phospho-Tau (Thr212,Ser214) (ATl00) mAb ThermoFisher MN1060): https://www.thermofisher.com/antibody/product/Phospho-Tau-Thr212-Ser214-Antibody-clone-AT100-Monoclonal/MN1060
Anti-CD31 mAb-EPR17259 (Abeam ab182981): https://www.abcam.com/products/primary-antibodies/cd31-antibody-epr17259-ab182981.html
Anti-HPl Rabbit polyclonal (Cell signaling 2616): https://www.cellsignal.com/products/primary-antibodies/hp1a-antibody/2616?_=1688511520867&Ntt=2616&tahead=true
Anti-DAPKl Antibody- Rabbit polyclonal (Cell signaling 3008): https://www.cellsignal.com/products/primary-antibodies/dapk1-antibody/3008?_=1688511543721&Ntt=3008&tahead=true
Anti Phospho-DAPKl (Ser289)- Rabbit polyclonal (ThermoFisher PA5-105873): https://www.thermofisher.com/antibody/product/Phospho-DAPK1-Ser289-Antibody-Polyclonal/PA5-105873
Goat anti-Rabbit IgG (H+L), HRP (ThermoFisher- 32460): https://www.thermofisher.com/antibody/product/Goat-anti-Rabbit-IgG-H-L-Secondary-Antibody-Polyclonal/32460
Rabbit anti-Mouse IgG (H+L), and HRP (1:2000; ThermoFisher Catalog # PA1-28568):
https://www.thermofisher.com/antibody/product/Rabbit-anti-Mouse-IgG-H-L-Secondary-Antibody-Polyclonal/PA1-28568

Eukaryotic cell lines

Policy information about cell lines and Sex and Gender in Research

Cell line source(s)
Primary Human Trophoblasts (detailed in Methods), HTR8 (ATCC) first trimester trophoblast cell line was obtained from Dr. Charles Graham, HUVEC were obtained from ATCC, ATG4 SUM159 and MCF-7, HEK293T were obtained from ATCC. Wild-type and Cdhl-/- MEFs were provided by Dr. Wenyi Wei. The first trimester HchEpC1b was generated in the manner outlined in the method. HchEpC1b is an immortalized HPV E6 and hTERT transfected EVT cell line. HchEpC1b-ATG4BC74A cells (ATG4BC74A) were generated by stable transfection with pMRX-IRES-puro-mStrawberry-ATG4BC74A, as stated in the method.

Authentication
All cell lines used in this study were authenticated by STR profile report.

Mycoplasma contamination
All cell lines in our laboratory were routinely tested for mycoplasma contamination and cells used in this study were mycoplasma free.

Commonly misidentified lines
(See ICLAC register)
No commonly misidentified cell lines were used.

Palaeontology and Archaeology

Specimen provenance

Specimen deposition

Dating methods

☐ Tick this box to confirm that the raw and calibrated dates are available in the paper or in Supplementary Information.

Ethics oversight

Note that full information on the approval of the study protocol must also be provided in the manuscript.

Animals and other research organisms

Policy information about studies involving animals; ARRIVE guidelines recommended for reporting animal research, and Sex and Gender in Research

Laboratory animals
Female 6-8 weeks-old hTau mice, Male hTau mice 8-12 weeks-old used for mating. All the mice were C57BL/6J background and housed in a specific pathogen-free facility under 12h light/dark cycle, temperature with 24±2°C, humidity between 30-70%, with access to food and water ad libitum.

Wild animals
The study did not involve wild animals.

Reporting on sex
In total, 118 hTau female mice were utilized in the study. Total 37 male hTau mice were used for mating purpose only. Male mice were randomly chosen for multiple mating purposes.

Field-collected samples
The study did not involve samples collected from field.

Ethics oversight
Animal studies were approved by Lifespan/Rhode Island Institutional Animal Care and Use Committee (IACUC; protocol number 505722), and performed in accordance with guidelines established by NIH Guide for the care and use of laboratory animals.

Note that full information on the approval of the study protocol must also be provided in the manuscript.

Clinical data

Policy information about clinical studies
All manuscripts should comply with the ICMJE guidelines for publication of clinical research and a completed CONSORT checklist must be included with all submissions.

Clinical trial registration

Study protocol

Data collection

Outcomes

Dual use research of concern

Policy information about dual use research of concern

Hazards

Could the accidental, deliberate or reckless misuse of agents or technologies generated in the work, or the application of information presented in the manuscript, pose a threat to:

No	Yes	
X	☐	Public health
X	☐	National security
X	☐	Crops and/or livestock
X	☐	Ecosystems
X	☐	Any other significant area

Experiments of concern

Does the work involve any of these experiments of concern:

No	Yes	
X	☐	Demonstrate how to render a vaccine ineffective
X	☐	Confer resistance to therapeutically useful antibiotics or antiviral agents
X	☐	Enhance the virulence of a pathogen or render a nonpathogen virulent
X	☐	Increase transmissibility of a pathogen
X	☐	Alter the host range of a pathogen
X	☐	Enable evasion of diagnostic/detection modalities
X	☐	Enable the weaponization of a biological agent or toxin
X	☐	Any other potentially harmful combination of experiments and agents

Plants

Seed stocks	
Novel plant genotypes	
Authentication	

ChIP-seq

Data deposition

☐ Confirm that both raw and final processed data have been deposited in a public database such as GEO.

☐ Confirm that you have deposited or provided access to graph files (e.g. BED files) for the called peaks.

Data access links<br>*May remain private before publication.*	
Files in database submission	
Genome browser session<br>(e.g. UCSC)	

Methodology

Replicates	
Sequencing depth	
Antibodies	
Peak calling parameters	
Data quality	
Software	

Flow Cytometry

Plots

Confirm that:

- [ ] The axis labels state the marker and fluorochrome used (e.g. CD4-FITC).
- [ ] The axis scales are clearly visible. Include numbers along axes only for bottom left plot of group (a 'group' is an analysis of identical markers).
- [ ] All plots are contour plots with outliers or pseudocolor plots.
- [ ] A numerical value for number of cells or percentage (with statistics) is provided.

Methodology

Sample preparation

Instrument

Software

Cell population abundance

Gating strategy

- [ ] Tick this box to confirm that a figure exemplifying the gating strategy is provided in the Supplementary Information.

Magnetic resonance imaging

Experimental design

Design type

Design specifications

Behavioral performance measures

Imaging type(s)

Field strength

Sequence & imaging parameters

Area of acquisition

Diffusion MRI ☐ Used ☐ Not used

Preprocessing

Preprocessing software

Normalization

Normalization template

Noise and artifact removal

Volume censoring

Statistical modeling & inference

Model type and settings

Effect(s) tested

Specify type of analysis: ☐ Whole brain ☐ ROI-based ☐ Both

Statistic type for inference

(See Eklund et al. 2016)

Correction

Models & analysis

n/a	Involved in the study
☐	☐ Functional and/or effective connectivity
☐	☐ Graph analysis
☐	☐ Multivariate modeling or predictive analysis

Functional and/or effective connectivity

Graph analysis

Multivariate modeling and predictive analysis

This checklist template is licensed under a Creative Commons Attribution 4.0 International License, which permits use, sharing, adaptation, distribution and reproduction in any medium or format, as long as you give appropriate credit to the original author(s) and the source, provide a link to the Creative Commons license, and indicate if changes were made. The images or other third party material in this article are included in the article's Creative Commons license, unless indicated otherwise in a credit line to the material. If material is not included in the article's Creative Commons license and your intended use is not permitted by statutory regulation or exceeds the permitted use, you will need to obtain permission directly from the copyright holder. To view a copy of this license, visit http://creativecommons.org/licenses/by/4.0/
